# Identification of transposable elements and satellite DNA in the Neotropical species *Drosophila amaguana* from the Ecuadorian Andean Forests

**DOI:** 10.1371/journal.pone.0337390

**Published:** 2025-12-10

**Authors:** Manuel Alejandro Coba-Males, Simon Orozco-Arias, Romain Guyot, Doris Vela

**Affiliations:** 1 Facultad de Hábitat, Infraestructura y Creatividad, Pontificia Universidad Católica del Ecuador, Quito, Ecuador; 2 Laboratorio de Genética Evolutiva, Facultad de Ciencias Exactas, Naturales y Ambientales, Pontificia Universidad Católica del Ecuador, Quito, Ecuador; 3 Department of Computer Sciences, Universidad Autónoma de Manizales, Manizales, Colombia; 4 Centre for Research in Agricultural Genomics, CRAG (CSIC-IRTA-UAB-UB), Campus UAB, Cerdanyola del Vallès, Barcelona, Spain; 5 Institut de Recherche pour le Développement (IRD), UMR DIADE, CIRAD, Université de Montpellier, Montpellier, France; 6 Department of Electronics and Automation, Universidad Autónoma de Manizales, Manizales, Colombia; University of Bari: Universita degli Studi di Bari Aldo Moro, ITALY

## Abstract

Genome size variation in eukaryotic species is largely influenced by repetitive DNA sequences such as transposable elements (TEs), simple repeats, and satellite DNAs (satDNAs), which do not necessarily correlate with organismal complexity. In insects, TEs are crucial to evolutionary processes and are correlated with variations in genome size. In this study, we describe, for the first time, the mobilome and satellitome of *Drosophila amaguana*, an Ecuadorian Neotropical species with a large, unexplored genome size, to assess the contribution of these repetitive DNA sequences to its genome composition. Using a draft genome assembly of approximately 455.5 Mb, generated from Illumina short-read sequences obtained from 10 wild specimens of *D. amaguana* collected at the Refugio de Vida Silvestre Pasochoa, we employed a *de novo* approach to create a manually curated TE library of 737 consensus sequences. We identified 716 novel TE families that had not been previously described, 20 TEs previously characterized in other *Drosophila* species, and one DNA transposon previously described in the *Lepeophtheirus* genus. The total TE content in the *D. amaguana* genome was 21.54%, distributed as follows: 6.35% Helitrons (1 superfamily), 5.13% LTR retrotransposons (5 superfamilies), 3.63% TIRs (9 superfamilies), 3.61% LINEs (7 superfamilies), 1.17% MITEs, 0.94% Maverick, 0.67% PLE, 0.02% SINEs, and 0.01% DIRS. We also identified 11.8% of simple repeats. Additionally, we estimated the satDNA content using Illumina raw reads and identified 16 satDNA families, all unique to the *Drosophila* genus, which comprise 4.90% of the genome. Overall, our results based on short-read data suggest that the large genome size of *D. amaguana* may not be the consequence of a high amount of TEs or satDNAs. Instead, its large genome size could be attributed to other factors (e.g., noncoding DNA occupying substantial portions of the genome or a high percentage of duplicated genes) that remain to be determined or explored in future studies using long-reads to overcome short-reads limitations. These findings may currently offer valuable insights into the adaptative and evolutionary processes of the *mesophragmatica* species group in the Andean forests.

## Introduction

Transposable elements (TEs) are mobile genetic elements that can mobilize and create new copies in different regions of the genome, affecting the functions of surrounding sequences and modifying the genome’s structure and size [1]. Understanding the role and impact of TEs in eukaryotic genomes has been a major research focus. The presence of transposable elements raises important questions regarding their regulatory functions, which were first identified by Barbara McClintock, who discovered these elements and linked them to genome regulation [2]. TEs are classified into two main classes based on their transposition mechanism: Class I, or retrotransposons, which use an RNA intermediate, and Class II, or DNA transposons, which use only a DNA intermediate [3].

The high variation in genome size among eukaryotic organisms does not always correlate with organismal complexity or with gene numbers; instead, it could be influenced by the abundance of TEs content [4,5]. The genome can expand to a larger size when TEs increase their copy number and move within the host genome [6]. Nevertheless, other non-coding regions such as introns and highly repetitive DNA sequences may also be involved in the increase in genome size [7].

Insects have often been used to study transposons because of their key roles in the evolution of these organisms [8]. TEs are involved in the acquisition of insecticide resistance [9], climate adaptation [10], and various functions that promote genetic diversification [11]. Insects are considered major contributors to much of the available information about transposable elements [12]. In many Arthropoda genomes, TE content has been highly variable both within and across insect orders [12,13], even differing between species within the same order [14]. Numerous research projects have described TE content in various insect species, often demonstrating a positive correlation between genome size and TE content. For instance, *Belgica antarctica* (99 Mb) and *Locusta migratoria* (6.3 Gb) are two insect species with the smallest and largest known genomes among insects with characterized TE content, with TE content ranging from less than 1% [15] to as high as 76.57% [16]. In mosquitoes, the TE content is correlated with genome size [14,17]. For *Aedes aegypti* (1279 Mb) [18], the TE proportion (~ 60%) [19] was approximately three times higher than the TE content (19.55%) [17] reported for *Anopheles gambiae* (265 Mb) [20].

For over 40 years, the *Drosophila* genus has been one of the most common models used to address biological and genetic questions [21]. Studying mobile elements in this genus has been possible through inter-and intraspecific crosses between different species [22–28]. The abundance and activity of TEs are related to the host genotype [29] and bioclimatic conditions under which the species develops [29,30]. For example, *Drosophila melanogaster* (180 Mb) has approximately 15–20% TEs [31–33], including up to 146 different families from 165 consensus sequences [34], with a main distribution of their transposons in its centromeric heterochromatin [35]. However, African populations of *D. melanogaster* have a lower TE content than populations from other continents [36]. These findings have led to the identification of TE content in various *Drosophila* species, including 12.24% [37] in *D. simulans* (132.2 Mb) [38], 14.95% [37] in *D. virilis* (165.9 Mb) [38], 14.77% in *D. busckii* (135.7 Mb) [39], 45.58% in *D. ananassae* (231 Mb) [39], 34.34% in *D. willistoni* (235.5 Mb) [39], and 47.07% [29] in *D. suzukii* (268 Mb) [40].

*Drosophila amaguana* is a member of the *mesophragmatica* group, which was first described in 1957 [41]. This group includes endemic Neotropical species of the *Drosophila* genus from South America [42]. Additionally, in Ecuador, species of the *mesophragmatica* group inhabit the inter-Andean valleys as well as high-altitude Andean forests [43,44]. Our aim was to identify for the first time the content of transposable elements (TEs) and satellite DNAs (satDNAs) in *D. amaguana* by sequencing and assembling a comprehensive genome using Illumina short reads. This species had a record-sized assembled genome, ~ 455.5 Mb, providing a unique opportunity to explore and understand the role of these TEs in its genomic structure. This study constitutes an important step toward explaining the composition and organization of this large genome.

## Materials and methods

### Genomic data, sequencing and assembling

A natural population of *D. amaguana* [43] was collected from Refugio de Vida Silvestre Pasochoa. The Pasochoa Forest is located in the Pichincha province (Ecuador), and the collection area is situated at an altitude of 3,310 meters above sea level (m.a.s.l) (0°26’00” S, 78°29’00” W). Genomic DNA (gDNA) from a pooled sample of 10 individuals (5 males and 5 females) was extracted using the protocol described by Piñol et al. [45]. A NanoDrop™ 2000 ThermoScientific spectrophotometer was used to evaluate the quantity and quality of the extracted gDNA: 8,754 ng/μL and 2.16 A_260_/A_280_ ratios, respectively. The TruSeq DNA PCR library (Illumina Inc., San Diego, CA, USA) was prepared for sequencing on an Illumina NovaSeq 6000 platform with paired-end reads (2 × 150 bp). First, the raw read data were processed, and their quality was assessed using FastQC version 0.12.1 [46]. The FastQC report corresponding to the paired-end raw reads is provided in S1 File. Since the mean quality score across all bases was above Q25 and no adapter sequences were detected, no trimming was performed, allowing the reads to be used as obtained for downstream analyses. Subsequently, a *de novo* scaffold-level assembly for *D. amaguana* was performed using Maryland Super Read Cabog Assembler (MaSuRCA) version 4.0.0 (https://github.com/alekseyzimin/masurca) [47] using the previously generated paired-end sequences. Quality metrics and gene prediction were assessed using Python script, assembly_stats version 0.1.4 (https://github.com/MikeTrizna/assembly_stats) [48], and Benchmarking Universal Single-Copy Orthologs v.5.8.3 (BUSCO) [49], respectively. BUSCO was run using the diptera_odb12 lineage dataset (5,067 genes from 76 genomes) and Augustus as gene predictors in the optional parameters. Finally, we used the Redundans pipeline version 2.01 (https://github.com/Gabaldonlab/redundans) [50], with default parameters, to evaluate whether the *D. amaguana* genome assembly exhibited high redundancy due to duplicated contigs. This analysis was performed without applying any modifications to the original assembly and served as a validation step for its use in all subsequent analyses.

This study was performed using the permit MAE-DNB-CM-2015–0030 assigned by the Ministerio del Ambiente, Agua y Transición Ecológica (MAATE) of Ecuador.

### Transposable elements *de novo* identification

To generate a non-redundant *de novo* TEs library, we conducted an initial exploration using three different bioinformatic tools to analyze the genome sequence of *D. amaguana*. The first pipeline used was the Extensive *de novo* TE Annotator (EDTA) (v.2.1.0; https://github.com/oushujun/EDTA) [51], which was run with default parameters to automatically execute the entire pipeline and compile a final *de novo* TE library. The *D. amaguana* genome was analyzed using RepeatModeler (v.2.0.3; http://www.repeatmasker.org/RepeatModeler/) [52] and RepeatMasker (v.4.1.5; https://www.repeatmasker.org/RepeatMasker/) [53] maintaining all default parameters while also adding the -LTRStruct parameter to build another *de novo* TE library. Finally, the *D. amaguana* genome was analyzed using the default parameters of reasonaTE (https://github.com/DerKevinRiehl/transposon_annotation_reasonaTE), a transposon annotation tool part of the TransposonUltimate package (https://github.com/DerKevinRiehl/TransposonUltimate) [54]. This analysis provided a FASTA file containing all TE copies identified in the genome. These programs allowed us to perform an initial verification of the TE composition in the *D. amaguana* genome.

### Manual curation of TE libraries and TEs annotation

We manually checked the TE dataset obtained previously using each automatic *de novo* tool. First, we concatenated the TE library outputs from the EDTA and RepeatModeler into one FASTA file. The concatenated file from the *de novo* TE libraries generated by EDTA and RepeatModeler was then used for manual curation. We excluded the reasonaTE output because the pipeline did not generate a TE library; instead, it annotated all the TE copies. The curation process was carried out using the Manual Curator Helper tool (MCHelper) (https://github.com/GonzalezLab/MCHelper) [55] in the fully automatic mode. As a result, we obtained an automatically curated TE library. Then, we manually inspected each TE consensus sequence using the Manual Inspection Module from MCHelper, which uses plots generated by TE-Aid (version 1.0; https://github.com/clemgoub/TE-Aid) [56], as well as Multiple Sequence Alignment Plots from CIAlign [57], and other useful structural information such as terminal repeat length and coding domain presence. We inspected each TE consensus sequence and filtered out those that corresponded to false positives (i.e., simple repeats or chimeric sequences). The *D. amaguana* genome was masked using RepeatMasker v. 4.1.2-p1 [53] with the manually inspected TE library, including TE libraries from other *Drosophila* species obtained from the Berkeley Drosophila Genome Project (BDGP) dataset (https://www.fruitfly.org/) [58] and the Manual Curated TE library (MCTE) [34] applying the following parameters: -lib -gff -nolow -no_is -norna. We then used the OneCodeToFindThemAll script [59] in the genome annotation to defragment the copies. TEs annotation was analyzed using an in-house Python script (Dynamics_Dama.ipynb) to obtain information on TE copy frequency and size distribution at the superfamily level, as well as TE proportion content and landscape at the order level. Additionally, an in-house Bash script (TEcopies_sequences.sh), utilizing the convert2bed tool from BEDOPS version 2.4.41 [60] and BEDTools version 2.18 [61], was used to extract all TE copy sequences from the whole genome. All these analyses were performed considering the full element length, from start to end position, as defined in the TE annotation file. Both scripts are available at the Zenodo repository (https://doi.org/10.5281/zenodo.16782536). Finally, we ran the Tandem Repeat Finder (TRF) program (https://github.com/Benson-Genomics-Lab/TRF) [62] to identify an estimate of other simple repeats in the masked genome. All the analyses were performed using the manually curated TE library.

### TE landscape

To visualize a brief evolutionary TE history and explore the approximate insertion times of TEs –whether TE copies were inserted more recently or correspond to older copies with greater accumulated mutations– at the order level in the *D. amaguana* genome, we constructed a repeat landscape based on the TE annotation using the in-house Python script (Dynamics_Dama.ipynb). We obtained the divergence percentage directly from the RepeatMasker output, which was calculated relative to the consensus sequence used to annotate each copy. This divergence corresponds to the percentage of nucleotides that do not match the consensus sequence in the manually curated library and each TE copy found in the genome, and was calculated using the following formula:


$$\%\;\text{Divergence}=\frac{\text{Mismatches}}{\text{Matches}+\text{Mismaches}}$$

### Clustering analysis to identify satellite DNA (satDNA)

We used Illumina paired-end sequencing raw data from *D. amaguana* to perform *de novo* clustering analysis with RepeatExplorer2 [63] on the Galaxy platform [64] for unsupervised identification of satDNAs. Initially, we randomly extracted 20 million paired-end FASTQ files using the Seqtk tool version 1.4 (https://github.com/lh3/seqtk) and uploaded them to the RepeatExplorer Galaxy server (https://repeatexplorer-elixir.cerit-sc.cz/galaxy/). We then checked the read quality using the FastQC tool version 0.12.1 [46], and once again, we randomly subsampled 759,425 reads from each paired-end file. This corresponds to 0.5x of genome coverage. After, the “preprocessing of fastq paired-reads” tool included on the RepeatExplorer Galaxy allowed removing adapters and filtering reads, excluding those with more than 5% of its sequence in low-quality bases. We set the cutoff quality value at Q10 and trimmed the reads to 150 bp. Filtered paired-end files were concatenated into a single FASTA file, which was the input for the RepeatExplorer pipeline. We used the following parameters: REXdb = Metazoa version 3.0, queue = “basic”, advance settings = “yes”, use custom repeat database = “yes”, and by default the threshold for clustering was 90% and assembly was 55%. The results were downloaded for further inspection, considering at first stage only putative satDNA sequences with not less than 0.01% of the genome proportion. From the selected group, a second filter was applied to maintain only those clusters that complied with three out of the four parameters: C-value ≥ 0.8, P-value ≥ 0.7, satellite probability ≥ 0.1, and globular or ring graph layout. Finally, we compared the corresponding consensus of the putative satDNA sequences using BLASTN [65] with known sequences reported from other *Drosophila* species by De Lima & Ruiz-Ruano [66] and Silva et al. [67], as well as with sequences available in NCBI.

## Results

We obtained 43.2 Gb of data from Whole-Genome Sequencing (WGS) reads for *D. amaguana*. These data provided a genome coverage of 68.3x. We constructed a *de novo* draft genome assembly of *D. amaguana* with a genome size of 455,546,830 bp (~455.5 Mb). We refer to this assembly as a ‘draft genome’ because it is not fully assembled at the chromosome level. However, based on the assembly quality metrics assessing contiguity and completeness (Table 1), this genome can be considered of reasonably good quality and suitable for downstream analyses. The *D. amaguana* genome had a contig N_50_/L_50_ of 21,343/5,503 bp and scaffold N_50_/L_50_ of 28,854/4,218 bp, with a GC content of 38.38%.

**Table 1:** Summary statistics of the assembled *D. amaguana* genome.

Assembly	*D. amaguana*
Assembled genome size (bp)	455,546,830
*N* contigs	88,220
Contig N_50_ (kb)^a^	21.343
*N* scaffolds	75,476
Scaffold N_50_ (kb)^a^	28.854
Longest contig (Mb)	2.411
Longest scaffold (Mb)	2.415
BUSCO genome^b^	
Complete	98.7%
Single	16.5%
Duplicated	82.2%
Fragmented	0.8%
Missing	0.4%
Total BUSCO groups searched	5,067

^a^The length of the smallest contig such that, when combined with all contigs of equal or greater length, they account for at least 50% of the total assembly.

^b^Obtained using Augustus as a gene predictor.

Then, the assembled *D. amaguana* genome was evaluated using Redundans, revealing a low heterozygosity level of 0.03%, represented by only 1,004 contigs across the entire genome (Table S1 in S2 File). These results suggest that the initially estimated genome size was neither overestimated nor affected by duplicated fragments in our assembly, supporting its suitability for subsequent analyses.

### An initial insight of *de novo* TEs identified in *D. amaguana*

Three different tools were used for the *de novo* identification of TEs and to estimate their composition within the *D. amaguana* genome. The non-curated results from EDTA showed 15.77% total TEs in the entire genome, 7.91% in retrotransposons, and 7.86% in DNA transposons (Fig S1A in S3 File). On the other hand, using the non-curated results from RepeatModeler we obtained a *de novo* TE library that was masked in the genome with RepeatMasker, here, we identified 19.90% of total TEs. However, 10.71% of the TEs sequences were not classified. The corresponding percentages for retrotransposons and DNA transposons were 4.81% and 4.38%, respectively (Fig S1B in S3 File). Finally, with reasonaTE, we only reported 8.24% of TEs in the *D. amaguana* genome. This report indicated that 6.27% of the genome corresponded to DNA transposons and 1.97% to retrotransposons (Fig S1C in S3 File).

The initial TE genome composition at the order level in the *D. amaguana* genome differed, depending on the tool used (Table 2). EDTA annotated that long terminal repeat (LTR) and terminal inverted repeat (TIR) elements occupied 7.41%, and 6.48% of the assembled genome, respectively. Meanwhile, both long interspersed nuclear elements (LINEs) and Helitrons had contents of less than 1.5%. RepeatModeler showed the highest percentage of unclassified sequences and was the only tool to classify TE sequences into short interspersed nuclear elements (SINEs) and Penelope-like elements (PLEs), both comprising less than 1% of each. These elements were reported either by EDTA or reasonaTE, but at almost 0.01% or less. In addition, Helitrons predominated over others, followed by LTR, LINE, and TIR elements. However, the TE composition indicated low percentages (less than 3%) in all orders when using RepeatModeler. Finally, reasonaTE identified only three transposon orders. TIR elements were the most abundant group (6.03%) in the genome, while Helitrons had a proportion of only 0.20%. This value was the lowest when compared to reports using EDTA (1.37%) and RepeatModeler (2.79%).

**Table 2:** TE percentage in the *D. amaguana* genome using non-curated libraries from *de novo* tools.

Transposable Elements	EDTA (%)	RepeatModeler (%)	reasonaTE (%)
LTRs	7.41	2.15	1.94
PLEs	0.01	0.66	NR
LINEs	0.48	1.74	0.03
SINEs	NR	0.26	< 0.001
TIRs	6.48	1.59	6.03
Helitrons	1.37	2.79	0.20
Maverick	0.01	NR	NR
MITEs	NR	NR	0.04
Unclassified^a^	0.01	10.71	NR

NR: Non-reported by pipeline in the summary file.

^a^Unclassified elements correspond to sequences that cannot be assigned to any TE order.

In some cases, other orders for TEs were identified at very low percentages. The summary report from EDTA indicated the presence of mobile elements, such as PLE, Maverick, and unclassified non-LTR sequences, which represented 0.01% of the total genome. Neither RepeatModeler nor reasonaTE reported Maverick in their analysis. However, EDTA did not identify SINEs. This order was identified by RepeatModeler at 0.26% and reasonaTE at a minimal percentage. Moreover, reasonaTE was the only method to classify 0.04% of the genome, specifically as miniature inverted-repeat transposable elements (MITEs) (Table 2).

### Overview of the curated TE dataset

Manual curation of TE libraries enabled us to characterize the mobilome in the assembled *D. amaguana* genome more precisely. Using *de novo* TE libraries obtained using EDTA and RepeatModeler, we built a curated TE dataset for *D. amaguana*. This dataset included 737 consensus elements, of which 292 (39.62%) were classified as Retrotransposons (Class I) and 445 (60.38%) as DNA transposons (Class II). Moreover, only 21 consensus sequences complied with the 80-80-80 rule [3], which is similar to the previously described transposon sequences (Table S2 in S2 File). Of these, 20 sequences corresponded to transposons found in other *Drosophila* species including *D. melanogaster*, *D. hydei*, *D. mercatorum*, *D. willistoni*, *D. ananassae*, *D. virilis*, *D. persimilis*, *D. elegans*, and *D. bipectinata*. The remaining sequence has been previously reported in *Lepeophtheirus salmonis* (Siphonostomatoida: Caligidae). Therefore, the 716 consensus sequences could be catalogued as potentially novel TE families that have never been described before.

Genome annotation was carried out with this own curated TE dataset, including other *Drosophila* TE libraries, such as the BDGP dataset [58] and the MCTE library [34]. Subsequently, 942 TEs were annotated in the *D. amaguana* genome, with approximately 53.18% corresponding to Class II elements and 46.82% to Class I elements (Table 3). Among these, 226 corresponded to known families. Furthermore, we identified 12 defined superfamilies of retrotransposons (LTR: *Gypsy*, *Copia*, *Bel-Pao*, *LARD*, *TRIM*; and LINE: *CR1*, *Jockey*, *I*, *R1*, *R2*, *RTE*, *Loa*) and 11 superfamilies of DNA transposons (TIR: *hAT*, *P*, *Tc1-Mariner*, *Pogo*, *PiggyBac*, *CMC*, *MULE-NOF*, *Transib*, *Merlin*; Helitron: *Helitron*; and Maverick: *Maverick*) (see more details in Table 3).

**Table 3:** Summary of TE annotation using the manually curated TE dataset for *D. amaguana*.

Class	Order	Superfamily	Annotated contigs^a^	TEs annotated	TE known families	New annotated TE
Class I	LTR	LTR^b^	11,367	41	7	34
	LTR	*Gypsy*	11,468	201	74	127
	LTR	*Copia*	490	22	8	14
	LTR	*Bel-Pao*	2,187	23	11	12
	LTR	*LARD*	4,067	4	1	3
	LTR	*TRIM*	2,072	4	2	2
	LINE	LINE^b^	7,682	21	9	12
	LINE	*CR1*	3,944	21	1	20
	LINE	*Jockey*	2,968	55	33	22
	LINE	*I*	814	9	3	6
	LINE	*R1*	4,029	22	11	11
	LINE	*R2*	103	2	1	1
	LINE	*RTE*	58	3	0	3
	LINE	*Loa*	21	2	2	0
	SINE	SINE^b^	535	4	1	3
	DIRS	DIRS^b^	540	1	0	1
	PLE	PLE^b^	5,700	6	1	5
	Total Class I		58,045	441	165	276
Class II	TIR	TIR^b^	20,061	192	18	174
	TIR	*hAT*	5,194	16	1	15
	TIR	*P*	630	6	6	0
	TIR	*Tc1-Mariner*	5,846	42	14	28
	TIR	*Pogo*	24	1	1	0
	TIR	*PiggyBac*	160	2	1	1
	TIR	*CMC*	686	1	0	1
	TIR	*MULE-NOF*	3	1	1	0
	TIR	*Transib*	227	11	6	5
	TIR	*Merlin*	82	1	0	1
	MITE	MITE^b^	12,241	173	4	169
	Helitron	*Helitron*	24,918	53	9	44
	Maverick	*Maverick*	5,337	2	0	2
	Total Class II		75,409	501	61	440
	Total		35,763^c^	942	226^d^	716

^a^Number of unique contigs in which at least one TE sequence was annotated.

^b^These TEs could not be classified at a level deeper than the order level and therefore do not correspond to specific superfamilies.

^c^This corresponds to the unique total annotated contigs in the entire genome.

^d^21 families correspond to the manually curated TE consensus library for *D. amaguana*, which had homology with TEs families previously described in other *Drosophila* species (16 retrotransposons and 4 DNA transposons). In addition, one family of DNA transposons showed similarity to a previously described TE sequence from a species in the *Lepeophtheirus* genus.

The dataset, including the manually curated transposable element (TE) library with consensus sequences for *D. amaguana*, genomic TE annotations, and the sequences of all TE copies, is available on Zenodo: https://doi.org/10.5281/zenodo.16782536.

Of the 737 consensus elements, we further classified the TEs based on their potential autonomy. We identified 150 autonomous TEs (Table 4), indicating their potential to be mobilized. In contrast, 587 TEs were identified as fragmented remnants (Table 4), lacking the essential features for autonomous transposition, such as intact ORFs or terminal sequences. This classification was based on the decision tree described in the supplemental materials of the MCHelper tool [55], which is crucial for distinguishing between potentially functional and non-functional transposons, allowing us to better understand the mobilization potential of the *D. amaguana* genome.

**Table 4:** Number of autonomous and non-autonomous transposable element (TE) orders identified in the *D. amaguana* genome using the manually curated TE library.

Transposable Elements	Autonomous	Non-Autonomous	Total
LTRs	13	190	203
LINEs	58	22	80
SINEs	0	3	3
DIRS	0	1	1
PLE	0	5	5
TIRs	28	200	228
Helitron	47	123	170
Maverick	0	2	2
MITEs	4	41	45
TOTAL	150	587	737
TOTAL (%)	20.35	79.65	100

### TEs distribution along the entire genome of *D. amaguana*

**Fig 1:**
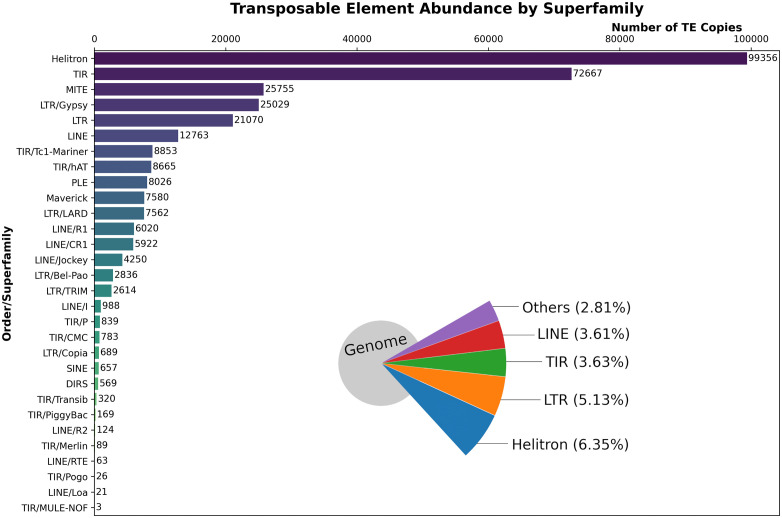
Abundance and genomic occupancy of transposable elements in *D. amaguana* based on annotations from a manually curated TE library. Each bar represents the total frequency of all copies annotated in the genome of *D. amaguana*. The names of the order/superfamilies are represented on the y-axis, whereas the frequency of the number of copies is represented on the x-axis. Pie chart showing the genomic occupancy percentages of each transposable element (TE) order in the assembled genome. The pie chart illustrates the relative contribution of Helitrons (6.35%), LTR elements (5.13%), TIR elements (3.63%), LINEs (3.61%), and others –including SINEs, DIRS, PLE, MITEs, and Maverick elements– (2.81%) to the total genome assembly.

We reported that, at the TE order level, Helitrons were the most abundant TEs in the entire genome, representing 30.64% of all TE copies (Fig 1). This corresponded to 99,356 copies and was represented by the only superfamily known to date, Helitrons (Table S3 in S2 File). SINEs and DIRS had the lowest number of copies (657 and 569, respectively) (Table S3 in S2 File). Furthermore, the remaining TE copies were distributed among the four orders (TIRs, LTRs, LINEs, and MITEs). TIRs elements were predominant, with 32,614 copies more than LTRs, 62,263 copies more than LINEs, and approximately 66,659 copies more than MITEs (Table S3 in S2 File). This makes them the second-most abundant TE order in *D. amaguana*. Among the TIR transposons, the *Tc1-Mariner* and *hAT* superfamilies had an important number of copies, representing 18.96% of all TIR copies. However, the largest number of copies of TIRs corresponded to TEs, which could not be classified at the superfamily level within this order, totaling 72,667 copies (Fig 1 and Table S4 in S2 File). The *Gypsy* superfamily contributed 25,029 copies that almost corresponded to at least half of the copies in the LTR retrotransposons, whereas the *CR1*, *Jockey*, and *R1* superfamilies similarly dominated the order of LINEs (Table S4 in S2 File). On the other hand, we found that the *Loa*, *RTE*, *MULE-NOF*, *Merlin*, and *Pogo* superfamilies had fewer than 100 copies, while the *PiggyBac* and *R2* superfamilies had fewer than 200 copies (Fig 1 and Table S4 in S2 File). In both cases, this represented a genome contribution less than or equal to 0.01%.

All superfamilies exhibited a wide variability in the size distribution of their TEs copies. For example, many copies of the DIRS and SINEs orders and the *Loa* superfamily were smaller than 500 bp, as well as some copies from the *CMC*, *MULE-NOF*, and *Pogo* superfamilies, which were less than approximately 200 bp (Figs S2 and S3 in S3 File). From a general perspective of the order level within LTRs and LINEs, most copies ranged from approximately 1 kb to 5 kb, with some *Gypsy* copies reaching up to 7.5 kb (Figs S2 and S3 in S3 File). Additionally, this size range was similar to that of the *Helitron* and TIRs copies, although for TIRs, a distribution starting from 500 bp was common (Figs S2 and S3 in S3 File). Finally, the *Maverick* superfamily had a few copies with large sizes, reaching up to > 8 kb, while the size of the MITEs was concentrated in a range of up to 3 kb (Figs S2 and S3 in S3 File).

### Repeat landscape of transposable elements

**Fig 2:**
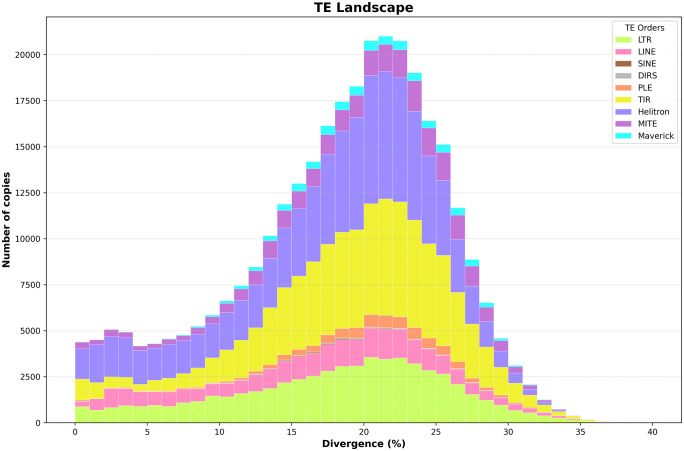
TE landscape of the *D. amaguana* genome. The landscape displays the distribution of full-length transposable elements (TEs) copies across divergence percentages, as a proxy for their relative age in the genome of *D. amaguana*. The x-axis represents the sequence divergence percentage of each TE copy from its consensus sequence, and the y-axis indicates the number of copies per TE order.

The TE landscape illustrates the frequency at which full-length TE copies from each order were found at different divergence percentages compared to their consensus sequences from the manually curated TE library. A sustained increase in TE activity was observed at divergence percentages ranging from approximately 15% to 25%, with frequencies between 10,000 and 20,000 TE copies across the entire *D. amaguana* genome (Fig 2). This increase mainly involved TE copies from Helitron, TIR, and LTR orders, which may explain why these orders show a higher number of copies in the genome. Moreover, this pattern may reflect ancient transposition events, suggesting that many TEs have not been recently mobilized within the *D. amaguana* genome. Additionally, the peak of activity around 20% to 22% divergence could indicate a burst of transposition involving not only the aforementioned orders but also PLE and Maverick elements (Fig 2), which appear inactive in recent evolutionary time, as they show no distinguishable copies at lower divergence levels.

### *De novo* estimation of satDNAs in *D. amaguana*

Automatic annotation from RepeatExplorer analysis retrieved 65,528 clusters grouped into 65,514 superclusters. Among these, 23 clusters were identified as putative satDNAs, with four having high confidence and 17 having low confidence. After the examination, filtering, and final annotation of each putative satDNA, the number of confirmed satDNAs decreased to 16 (Table S5 in S2 File). Each cluster of these 16 satDNAs exhibited a globular or ring shape in the graph layout. In addition, they had C (connected component index) and P (pair completeness index) indices close to 1. These are key parameters for maintaining the identification of clusters as typical sequences of true satellites.

None of these satDNAs have been described to date. Moreover, BLASTN results did not show similarities with any satDNA families previously reported in 37 *Drosophila* species from the *Sophophora* and *Drosophila* subgenera [66] and/or with *Drosophila* species from the *montium* group [67]. Additionally, a homology search of the identified satDNAs did not yield any similarities with other satDNA sequences deposited in NCBI. Therefore, the consensus sequences for these 16 satDNAs were catalogued as new families, named DamaSat01–97 to DamaSat16–1260, according to the nomenclature rules proposed by Ruiz-Ruano et al. [68]. These complete sequences are available in S4 File.

DamaSat01–97 is a satellite with a total repeat length of 97 bp and the highest %AT content of 76.29%. Moreover, its contribution to the *D. amaguana* genome was 1.75%, which is a unique DNA satellite with a proportion higher than 1% in the genome. It is likely that DamaSat02–14 and DamaSat03–15 may be considered satellites with a medium proportion within the genome at 0.97% and 0.78%, respectively. The array lengths of these sequences were 14 bp and 15 bp, respectively, and they exhibited a similar %AT content of approximately 60%. Despite the consensus length of 14 out of 16 satellites up to 1.2 kb, the total array length for two sequences (DamaSat05–3461 and DamaSat11–6402) was significantly longer, approximately 3.4 kb and 6.4 kb, respectively. These sequences exhibited high satellite probabilities of 0.979, C and P indices close to 0.99, and a clear ring-graph layout. These characteristics are consistent with those typically associated with satDNAs, and the total array lengths support their classifications. Finally, we estimated the amount of satDNAs present in the raw reads of *D. amaguana* to be 4.90%.

### Contribution of the repetitive DNA within the *D. amaguana* genome

**Fig 3:**
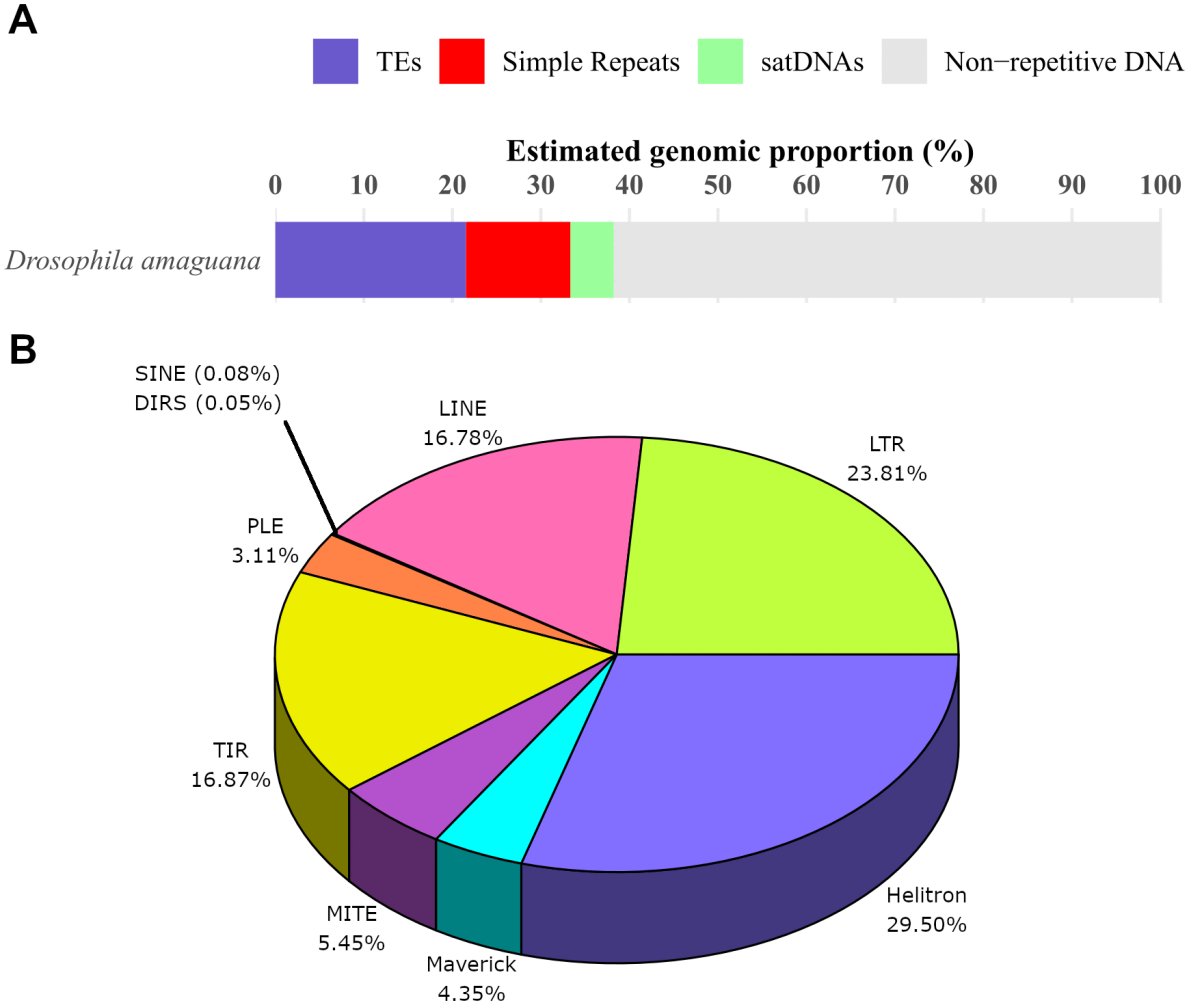
Repetitive DNA. **(A)** Estimated percentage of repetitive DNA comprising the large genome of *D. amaguana* calculated using *de novo* assembled genome. The satDNA percentage is an estimate based on raw reads **(B)** Percentage of each transposable element order relative to the total TE content annotated in the assembled *D. amaguana* genome after manual curation.

Our analysis revealed that 61.76% of the assembled genome of *D. amaguana* consisted of nonrepetitive DNA (Fig 3A). The total content of repetitive DNA present in the large genome assembly of *D. amaguana* included transposable elements, satellite DNAs, and simple repeats (Fig 3A). Of these repetitive DNA, TEs were the most abundant type of repetitive sequences in this genome, accounting for 21.54%, which corresponded to approximately 98.1 Mb. Helitrons were the most abundant, with 29.50% of the total TEs, followed by LTRs (23.81%), TIRs (16.87%), and LINE (16.78%), while the rest of the TE orders contributed 13.03% (Fig 3B). Other types of repetitive DNA in the assembled genome of *D. amaguana* included simple repeats (11.80%). In contrast, satellite DNA may represent 4.90% of the genome based on raw reads.

## Discussion

The aim of this study was to provide an initial overview of the repetitive elements –mainly transposable elements and satellite DNA– in *D. amaguana*, a neotropical species that currently possesses the largest *de novo* draft genome (~455.5 Mb) reported in the genus. Previously, *Drosophila cyrtoloma* was considered to have the largest genome size (~401 Mb) in the *Drosophila* genus, based on cytometric estimates [69,70]. Our findings offer insights into the composition and potential contribution of repetitive elements to the genome expansion observed in *Drosophila*.

### Transposable elements within the *D. amaguana* genome

Genome size has been associated multiple times with TE content. This has led to the consideration that TEs are key actors in insect genomes [8], not only by their contribution to genomic structures, but also by their role in the evolution and adaptation of the species [11]. Within the insect order Diptera, TE content ranges from 1% to 55% [14]. In *Drosophila* species, TE content varies from 2.7% to 25% [71], even reaching up to 40% in species such as *D. ananassae* [14]. We assessed the percentage of TEs in *D. amaguana* and estimated that the mobilome corresponded to 21.54%. To our knowledge, this is the first report of TEs in a species from the *mesophragmatica* group, which has a large genome size (~455.5 Mb). Moreover, it could provide new insights into genome organization and potentially offer information on the role of these sequences in the adaptive radiation of Neotropical groups.

The best approach for *de novo* transposable element discovery and identification is still an active and unresolved research question [71]. The diversity of software and methodologies can generate slightly different results for the same species [72]. Although each pipeline has a defined workflow or shares tools to identify TEs, developers can define different approaches. Our preliminary results obtained through an automatic *de novo* TE annotation with three different tools showed that the TE content in *D. amaguana* ranged from 8.24% to 19.90%. This particularity has also been observed in *D. willistoni,* where two different bioinformatics tools estimated 9% and 16% of TEs in its genome [71], and in the genome of *D. ananassae,* which showed 12% and 20% of TEs when using the same tool but considering a reference library or with a *de novo* approach, respectively [71].

Although the development of tools for identifying and annotating mobile elements has been growing, characterizing the mobilome in a genome is still a challenge owing to the high complexity of repetitive DNA [73]. However, the raw TE libraries generated by *de novo* tools may contain redundant, fragmented, or false-positive TE consensus sequences [74]. For this reason, it must always be manually checked to ensure that the sequences are accurate and truly belong to TEs [55,56]. The manual curation of the *D. amaguana* mobilome included raw TE libraries from EDTA and RepeatModeler, excluding the results from reasonaTE because its estimated percentage was low and did not automatically provide a TE consensus library. Therefore, the analysis of *D. amaguana* identified 737 elements, 716 of which were discovered for the first time in *Drosophila*. This suggests that these sequences may represent novel TE families.

The TE annotation of the entire genome of *D. amaguana* performed with our curated library but also including curated libraries from other species of *Drosophila*, allowed us to gain insight into the different TEs types that compose this genome. One of the most relevant findings was the presence of the *P* superfamily, as the transposition of *P* elements has been associated for many years with hybrid dysgenesis events [27]. Within the *mesophragmatica* group, tropical species reported in Brazil, Colombia, and Chile, such as *D. gaucha*, *D. gasici*, *D. pavani*, and *D. viracochi*, did not show the presence of *P* elements [75,76]. Therefore, this finding would help to understand the role and origin of this superfamily in the host genome and the adaptive process, because the emergence of this class of TEs has been associated with horizontal transfer events [77]. It can occur owing to geographic, temporal, and ecological factors [78].

However, the detection of *MULE-NOF* and *CMC* superfamilies, although they comprise less than 0.01% of the genome, is still noteworthy. Despite some reports of *Muta1* elements identified in insects such as *Aedes aegypti* [79], characterization of the *MULE* superfamily in other species is quite rare [80], as studies have focused more on fungi and plants [81]. A similar situation has been observed in the *CMC* superfamily. Although there are studies on *CMC* elements in insects, such as *Mengenilla moldrzyki*, *Rhodnius prolixus*, and *Bombyx mori* [82], these findings are limited because most reports focus on plants, which can account for up to 10% of transposons in the genome [83].

Many insects are susceptible to invasion by the *Tc1-Mariner* superfamily [84]. Some families of this class of TEs have been shown to be autonomously functional in *Drosophila* [85], even in evolutionarily distant species, thereby introducing genomic novelty [86,87]. Mariner elements have been identified *in vitro* for *D. gasici*, *D. brncici*, and *D. viracochi* [76]. Thus, *D. amaguana* was not far behind because the *Tc1-Mariner* superfamily was the highest contributor within the TIR order for the classified superfamilies, with an abundance of 0.48%.

LTR retrotransposons are a widely represented class of TEs in *Drosophila* genomes. *D. yakuba*, *D. simulans*, *D. sechellia*, and *D. erecta* have 141 new LTR elements and 76 sequences homologous to TEs of *D. melanogaster* have been described [88]. In this study, we annotated 295 LTR retrotransposons in *D. amaguana* and identified 192 new elements. Within this order, *Gypsy* and *Copia* were the superfamilies with the highest (25,029) and lowest (689) number of copies, representing 2.69% and 0.09% of the whole genome composition, respectively. A low number of copies in the *Copia* superfamily has also been observed in the model species *D. melanogaster*, where it has been relatively number compared to other organism classes, barely between 10 and 100 copies [89]. However, this superfamily is widespread in nature owing to its potential transposition activity, and some of them have been involved in horizontal transfer events between *D. melanogaster* and *D. willistoni* [88,90]. The *Gypsy* superfamily is highly diversified and numerous in several *Drosophila* species, including *D. willistoni*, *D. ananassae*, *D. elegans*, and –with the exception of *D. erecta*– all the species in the *melanogaster* group have been reported to contain between 41 and 61 different *Gypsy* families [88]. This makes it the most abundant superfamily of LTR retrotransposons in species, such as *D. sechellia* (9.36%), *D. grimshawi* (9.77%), and *D. ananassae* (12.44%), which contain *Ty3/Gypsy* type elements [88]. Additionally, *D. brncici*, *D. pavani*, *D. viracochi*, and *D. gasici* (with three populations from Chile, Colombia, and Bolivia) have been reported to contain *Gypsy* retrotransposons in their genomes [76]. Homologous sequences of *Copia* retrotransposons have been found in *D. pavani*, but there is limited information about *Copia* retrotransposons in other species of the *mesophragmatica* group [91].

Petersen et al. [14], assessed 62 arthropod genome sequences of insects identifying that LTR retrotransposons are dominant in the Diptera order, DNA transposons are representatives in Hymenoptera, LINE retrotransposons are prevalent in Hemiptera, and SINE retrotransposons had the lowest contribution in all insect order. The mobilome of *D. incompta*, a neotropical species with restricted ecology, belongs to the *flavopilosa* group that depends on flowers for its development [92], indicating that 14.62% of the genome corresponds to TEs, with LTR, LINE retrotransposons, and Helitrons as the most representative groups [73]. Other study in the genome of *D. buzzatii* st-1 (8.43% of TEs in a genome of ~153 Mb), *D. buzzatii* j-19 (4.15% of TEs in a genome of ~146 Mb), and *D. mojavensis* (15.35% of TEs in a genome of 194 Mb) identified Helitrons as the most abundant group in all species [93]. LTR retrotransposons were the second order more abundant only in *D. mojavensis*, due to the presence of LINE elements in both D. buzzatii species, whereas TIR transposons are catalogued as the third group abundant in all of them [93]. Our results in *D. amaguana* showed that Helitrons (6.35%), followed by LTR (5.13%), TIR elements (3.63%), and LINE (3.61%) were the most abundant TEs in genome composition. The abundance of Helitron elements has also been observed in *D. virilis*, *D. miranda*, and *D. pseudoobscura* [14]. SINE retrotransposons contributed to only 0.02%, which is commonly observed in most dipterans, where SINEs contribute less than 1% [14], or in the whitefly species *Bemisia tabaci*, where SINEs were the least represented TEs at 0.14% [94]. Furthermore, despite many *Drosophila* genomes showing less diversity and abundance of MITEs compared to other mosquito species and plants [95], *D. amaguana* has 1.17% MITE, making them the four most abundant class of TEs.

Analyzing the abundance of TE in *D. melanogaster* has also been attractive to some researchers with a predominance of LTR retrotransposons, followed by LINE elements, and finally, TIR transposons [58,96]. A conservative approach suggests that trend in the rank abundance of TEs in these orders is conserved across genus [71]. Usually, Class I elements predominant over Class II [8]. Nevertheless, there are exceptions that do not adjust to this postulate, such as *D. erecta*, which has a higher abundance of TIR elements, *D. pseudoobscura*, which has lower abundance of LTR retrotransposons [71], and now *D. amaguana*. All these variations and differences are owing to a variety of host factors, such as the transposition rate of progenitors, and the rate at their copies are inactivated and become unable to transpose [5].

### TE landscape

Understanding the evolutionary dynamics of TEs is essential for comprehending when these sequences colonized the genome and how they contribute to expanding genome size. For this purpose, TE landscapes are very useful to visualize and characterize TE dynamics [73]. The graph groups TE copies based on the percentage divergences regarding their respective TE consensus sequence. Copies on the left of the graph correspond to recent copies, while those on the right correspond to more divergent copies compared to their consensus [97].

Almost all drosophilids within Diptera order, have shown a unimodal distribution with highest peaks corresponding to LTR retrotransposon as result of recent activity into the dipteran genomes or DNA transposons in ants [14]. In *D. amaguana*, the TE landscape shows a unimodal shape with an important TE activity ranging from 15% to 25% divergence. The TE activity refers to the active mobilization or continuous insertion of transposable elements in the genome, indicating that these elements are still active and dynamic. The main peak, defined between 20% and 22% sequence divergence, represents approximately 7–8% of the total number of Helitron copies. Furthermore, the occurrence of older transposition events involving Helitrons is well documented. For example, approximately 1% of the *D. melanogaster* genome consists of ancient Helitron remnants, while around 3% of the *D. yakuba* genome is composed of both autonomous and non-autonomous Helitrons, reflecting their historical activity within the genome.

In *D. melanogaster*, most LTR retrotransposons are considered relatively recent elements [98]. In contrast, in *D. amaguana*, several LTR copies (approximately 3,000–3,500) exhibited up to 20% sequence divergence from their consensus. This may suggest substantial TE activity, reflected by patterns indicative of older insertions, particularly for LTR retrotransposons and Helitrons. TIR elements also exhibit a strong signal at around 20–22% divergence, which may indicate a past burst of activity, although their apparent absence at lower divergence levels suggests they have not been recently active in *D. amaguana*. Such TE bursts may have resulted from successful habitat expansions, potentially linked to stress-induced disruption of protective mechanisms such as the piRNA pathway, or from interspecies contact [99]. For example, the well-known case of *P* element transfer from *D. willistoni* to *D. melanogaster* occurred after range overlap in South America; soon after, the *P* element rapidly spread across global *D. melanogaster* populations.

The low contiguity of the assembled genome may have hindered the detection of more recent insertions with low sequence divergence. However, given the limited genomic information available for *D. amaguana*, further analyses are needed to confirm or refine these observations.

### Satellite DNA as other repetitive DNA contributors

As well as mobile elements, other types of noncoding DNA, may also be key to understanding why some species have large genome sizes. Transposable elements and satellite DNA are the main driving force influencing the genome size of *Drosophila*; these repetitive sequences have shown a positive correlation with larger genomes sizes [100]. For example, both longer introns [101] and lengthier stretches of microsatellites [102] have been reported in *D. virilis* compared to *D. melanogaster*, which could help explain why the increased presence of non-coding and repetitive DNA causes the *D. virilis* genome to be approximately twice as large as that of *D. melanogaster* genome. Another observed case is *D. cyrtoloma* (401 Mb), the species currently known to have the largest genome within the *Drosophila* genus. Its large genome is due to at least ~70% satellite DNA content [69]. However, there are also findings where satDNAs are not the principal contributors of repetitive DNA and genome size [103], such as in *D. erecta* (153 Mb), where only 0.4% of its genome is composed of satDNA [104].

The class Insecta includes a wide range of satDNA families [68,105–108]. These sequences are involved in evolution, being unique to each species and showing high dynamism within repetitive DNA [11,109]. For instance, up to 16 families of satDNAs have been reported in *D. melanogaster* [110], between 2–14 families in species from the *montium* group [67]. We found 16 satDNA families in *D. amaguana*, corresponding to 4.90% of the genome. This proportion is similar to the 5% satDNA content reported in *D. simulans* (142 Mb) [104]. None of the satDNA sequences from *D. amaguana* showed homology with families reported in other species, probably due to underrepresented sequences in available databases.

The satDNAs in *Drosophila* are variable in terms of repeat array size, nucleotide composition, and genomic proportion [66]. The variation in repeat length among the satellites found in *D. amaguana* ranges from 6 bp (DamaSat04–6 and DamaSat09–6) to 6.4 kb (DamaSat11–6402), which, to the best of our knowledge, is the largest satDNA reported in insects. This is significantly larger than previously reported satDNAs in other species, such as *Chrysolina americana* (3664 bp) [111] and *Mahanarva* species (4228 bp) [112]. Most common satDNA lengths in the *Drosophila* genus range from <10 bp to 400 bp [113–115], with some even reaching up to 570 pb [66]. 11 out of 16 satDNA families in *D. amaguana* fall within this range, while three families (DamaSat05–3461, DamaSat11–6402, and DamaSat16–1260) had a consensus length greater than 1 kb. Monomer sizes exceeding 1 kb have also been described in other insect species, such as the *Vandiemenella* morabine grasshoppers [116], the ladybird beetle *Hippodamia variegata* [117], the beetle *Euchroma gigantea* [118], and the red flour beetle *Tribolium castaneum* [108], where repeat lengths can extend to several thousand base pairs [119].

### General overview of repetitive DNA content in *D. amaguana*

Initially, 21.54% of TEs reported in *D. amaguana* may seem low compared to other species with genome sizes up to three times smaller. For example, *D. melanogaster* has been reported to have 17.11% of TEs in an estimated genome size of 142.6 Mb [51]. This value was close to approximately 20% TE content reported for a 180 Mb genome [120], or the 19.3% reported by Mérel et al. [37]. Although this comparison is not intended as a definitive conclusion, it serves as a hypothesis based on preliminary data suggesting that the TE percentage may be relatively low. Nonetheless, it is important to acknowledge that, in many cases, there is a reported trend of a positive correlation between genome size and TE content, with larger genomes typically containing a higher proportion of TEs [8,121]. On the other hand, simple repeats content represented 11.80% of the genome, while satDNA content estimated using raw reads was 4.90%.

During the last two decades, multiple studies in *Drosophila* have contributed to new knowledge about the factors underlying genome organization. In this context, genome size in *Drosophila* has been approached from diverse perspectives. Ecological factors in the environments where species develop may be related to the genome size, as certain habitats could reduce the likelihood of horizontal transfer events involving TE families. For example, *D. grimshawi* was described as an island endemic species with low transposable element content [71]. Therefore, variation in TE content may result from adaptive responses to environmental stress during specific periods or within particular ecosystem [122]. This supports a hypothesis proposed years ago, suggesting that DNA content is often influenced by the environment in which an organism lives [123]. Other important contributors to genome size include chromosomal rearrangements [124], the presence of heterochromatic genes [125], and the role of other types of noncoding DNA [126,127]. Additionally, the percentage of duplicated genes observed in the assembled *D. amaguana* genome provides an opportunity to investigate the basis of this particularity in future studies. Duplication events in insects have been shown to play key roles in genome size variations [128], evolutionary innovative [129], phenotypic diversification [130,131].

However, it should also be noted that short-read assemblies often struggle to accurately resolve repetitive regions and highly similar sequences, leading to gaps, unassembled regions, fragmented contigs, collapsed repetitive sequences, and redundant or duplicated sequences in the final assembly. These issues can affect assembly accuracy and complicate downstream analyses, including gene prediction and the functional annotation of many genetic elements. Although the initial Redundans results suggested low heterozygosity and less than 2% duplicated contigs in our assembly, it is important to emphasize that the high level of gene duplication observed in the *D. amaguana* genome may or may not solely reflect biological reality. In addition to genuine gene duplications –which could be the focus of future specific studies in this species– technical limitations inherent to short-read sequencing may artificially inflate duplication estimates. Similarly, the estimated proportion of transposable elements (TEs) may also be underestimated, considering the generally positive correlation between TE content and genome size observed in other species or taxa. These results should therefore be interpreted with caution and will require confirmation and refinement using long-read sequencing technologies.

Further detailed research and deeper analyses may significantly help answer key questions about the larger genome size observed in *D. amaguana*. Expanding the study to include other individuals or populations [132], and applying third generation sequencing technologies to obtain long reads for generating a new genome assembly, could help address unresolved questions within the *mesophragmatica* group. These approaches may allow the detection of more complete sequences [133,134], overcoming current limitations not only to better characterize mobile elements [34,135], but also to provide a more accurate and comprehensive understanding of the biology of this Andean species of *Drosophila*. In particular, third-generation sequencing –which offers greater continuity and accuracy across complex genomic regions– will be essential to more reliably assess gene duplication, refine estimates of TE content, and ultimately clarify the true structure and composition of the *D. amaguana* genome.

In conclusion, our study showcases the first effort in the identification of repetitive DNA –including transposable elements (TEs) and satellite DNAs (satDNAs)– in Neotropical *Drosophila* species belonging to the *mesophragmatica* group that inhabit Andean forests in Ecuador. We identified 21.54% TEs and reported 11.80% simple repeats in the ~ 455.5 Mb draft genome assembly. In addition, we estimated the satDNA content using raw reads, which resulted in 4.90%. Our findings revealed the presence of all the TE orders, with a high prevalence of *Helitron*, *TIR*, *LTR* and *LINE* superfamilies. Moreover, we discovered 16 new satDNA families. The TE and satDNA content in *D. amaguana* do not appear to significantly contribute to its large genome size, as the TE proportion is similar to that of other *Drosophila* species with smaller genomes. However, these results provide valuable insights for future research on the role of mobile sequences and genome architecture in the adaptation and development of *D. amaguana*. They also represent a starting point for further analyses within the *mesophragmatica* group to better understand, among other things, the factors driving large genome sizes and evolutionary processes.

## Supporting information

S1 File FastQC report for paired-end raw reads of *Drosophila amaguana.* (PDF)

S2 File Supplementary Tables S1–S5. (PDF)

S3 File Supplementary Figures S1–S3. (PDF)

S4 File FASTA file of consensus sequences from the 16 satellite DNA (satDNAs) families identified in *D. amaguana.* (FASTA)

## References

[1] Kidwell MG. Evolution of hybrid dysgenesis determinants in Drosophila melanogaster. Proc Natl Acad Sci U S A. 1983;80(6):1655–9. doi: 10.1073/pnas.80.6.1655 6300863 PMC393661

[2] McClintock B. The origin and behavior of mutable loci in maize. Proc Natl Acad Sci U S A. 1950;36(6):344–55. doi: 10.1073/pnas.36.6.344 15430309 PMC1063197

[3] Wicker T, Sabot F, Hua-Van A, Bennetzen JL, Capy P, Chalhoub B, et al. A unified classification system for eukaryotic transposable elements. Nat Rev Genet. 2007;8(12):973–82. doi: 10.1038/nrg2165 17984973

[4] Suh A. Genome size evolution: small transposons with large consequences. Curr Biol. 2019;29(7):R241–3. doi: 10.1016/j.cub.2019.02.032 30939304

[5] Almojil D, Bourgeois Y, Falis M, Hariyani I, Wilcox J, Boissinot S. The structural, functional and evolutionary impact of transposable elements in Eukaryotes. Genes (Basel). 2021;12(6):918. doi: 10.3390/genes12060918 34203645 PMC8232201

[6] Wang J, Itgen MW, Wang H, Gong Y, Jiang J, Li J, et al. Gigantic genomes provide empirical tests of transposable element dynamics models. GPB. 2021;19(1):123–39. doi: 10.1016/j.gpb.2020.11.005 33677107 PMC8498967

[7] Hjelmen CE, Blackmon H, Holmes VR, Burrus CG, Johnston JS. Genome size evolution differs between Drosophila subgenera with striking differences in male and female genome size in Sophophora. G3. 2019;9(10):3167–79. doi: 10.1534/g3.119.400560 31358560 PMC6778784

[8] Maumus F, Fiston-Lavier A-S, Quesneville H. Impact of transposable elements on insect genomes and biology. Curr Opin Insect Sci. 2015;7:30–6. doi: 10.1016/j.cois.2015.01.001 32846669

[9] Chen S, Li X. Transposable elements are enriched within or in close proximity to xenobiotic-metabolizing cytochrome P450 genes. BMC Evol Biol. 2007;7(1):46. doi: 10.1186/1471-2148-7-46 17381843 PMC1852546

[10] González J, Karasov TL, Messer PW, Petrov DA. Genome-wide patterns of adaptation to temperate environments associated with transposable elements in Drosophila. PLoS Genet. 2010;6(4):e1000905. doi: 10.1371/journal.pgen.1000905 PMC2851572 20386746

[11] Cabral-de-Mello DC, Palacios-Gimenez OM. Repetitive DNAs: the ‘invisible’ regulators of insect adaptation and speciation. Curr Opin Insect Sci. 2025;67:101295. doi: 10.1016/j.cois.2024.101295 39521343

[12] Gilbert C, Peccoud J, Cordaux R. Transposable elements and the evolution of insects. Annu Rev Entomol. 2021;66:355–72. doi: 10.1146/annurev-ento-070720-074650 32931312

[13] Wu C, Lu J. Diversification of transposable elements in arthropods and its impact on genome evolution. Genes (Basel). 2019;10(5):338. doi: 10.3390/genes10050338 31064091 PMC6562904

[14] Petersen M, Armisén D, Gibbs RA, Hering L, Khila A, Mayer G, et al. Diversity and evolution of the transposable element repertoire in arthropods with particular reference to insects. BMC Evol Biol. 2019;19(1):11. doi: 10.1186/s12862-018-1324-9 30626321 PMC6327564

[15] Kelley JL, Peyton JT, Fiston-Lavier A-S, Teets NM, Yee M-C, Johnston JS, et al. Compact genome of the Antarctic midge is likely an adaptation to an extreme environment. Nat Commun. 2014;5:4611. doi: 10.1038/ncomms5611 25118180 PMC4164542

[16] Li X, Mank JE, Ban L. The grasshopper genome reveals long-term gene content conservation of the X Chromosome and temporal variation in X Chromosome evolution. Genome Res. 2024;34(7):997–1007. doi: 10.1101/gr.278794.123 39103228 PMC11368200

[17] Melo ES de, Wallau GL. Mosquito genomes are frequently invaded by transposable elements through horizontal transfer. PLoS Genet. 2020;16(11):e1008946. doi: 10.1371/journal.pgen.1008946 33253164 PMC7728395

[18] Matthews BJ, Dudchenko O, Kingan SB, Koren S, Antoshechkin I, Crawford JE, et al. Improved reference genome of Aedes aegypti informs arbovirus vector control. Nature. 2018;563(7732):501–7. doi: 10.1038/s41586-018-0692-z 30429615 PMC6421076

[19] Daron J, Bergman A, Lopez-Maestre H, Lambrechts L. Atypical landscape of transposable elements in the large genome of Aedes aegypti. bioRxiv. 2024:2024.02.07.579293. doi: 10.1101/2024.02.07.579293

[20] Sharakhova MV, Hammond MP, Lobo NF, Krzywinski J, Unger MF, Hillenmeyer ME, et al. Update of the Anopheles gambiae PEST genome assembly. Genome Biol. 2007;8(1):R5. doi: 10.1186/gb-2007-8-1-r5 17210077 PMC1839121

[21] Markow TA. The secret lives of Drosophila flies. eLife. 2015;4. doi: 10.7554/elife.06793 PMC4454838 26041333

[22] Vela D, Fontdevila A, Vieira C, García Guerreiro MP. A genome-wide survey of genetic instability by transposition in Drosophila hybrids. PLoS One. 2014;9(2):e88992. doi: 10.1371/journal.pone.0088992 24586475 PMC3930673

[23] Romero-Soriano V, Burlet N, Vela D, Fontdevila A, Vieira C, García Guerreiro MP. Drosophila females undergo genome expansion after interspecific hybridization. Genome Biol Evol. 2016;8(3):556–61. doi: 10.1093/gbe/evw024 26872773 PMC4824032

[24] Carnelossi EAG, Lerat E, Henri H, Martinez S, Carareto CMA, Vieira C. Specific activation of an I-like element in Drosophila interspecific hybrids. Genome Biol Evol. 2014;6(7):1806–17. doi: 10.1093/gbe/evu141 24966182 PMC4122939

[25] Lopez-Maestre H, Carnelossi EAG, Lacroix V, Burlet N, Mugat B, Chambeyron S, et al. Identification of misexpressed genetic elements in hybrids between Drosophila-related species. Sci Rep. 2017;7:40618. doi: 10.1038/srep40618 28091568 PMC5238404

[26] Vieira C, Fablet M, Lerat E, Boulesteix M, Rebollo R, Burlet N, et al. A comparative analysis of the amounts and dynamics of transposable elements in natural populations of Drosophila melanogaster and Drosophila simulans. J Environ Radioact. 2012;113:83–6. doi: 10.1016/j.jenvrad.2012.04.001 22659421

[27] Bingham PM, Kidwell MG, Rubin GM. The molecular basis of P-M hybrid dysgenesis: the role of the P element, a P-strain-specific transposon family. Cell. 1982;29(3):995–1004. doi: 10.1016/0092-8674(82)90463-9 6295641

[28] Daniels SB, Peterson KR, Strausbaugh LD, Kidwell MG, Chovnick A. Evidence for horizontal transmission of the P transposable element between Drosophila species. Genetics. 1990;124(2):339–55. doi: 10.1093/genetics/124.2.339 2155157 PMC1203926

[29] Mérel V, Gibert P, Buch I, Rodriguez Rada V, Estoup A, Gautier M, et al. The worldwide invasion of Drosophila suzukii is accompanied by a large increase of transposable element load and a small number of putatively adaptive insertions. Mol Biol Evol. 2021;38(10):4252–67. doi: 10.1093/molbev/msab155 34021759 PMC8476158

[30] Arnault C, Dufournel I. Genome and stresses: reactions against aggressions, behavior of transposable elements. Genetica. 1994;93(1–3):149–60. doi: 10.1007/BF01435247 7813912

[31] Quesneville H, Bergman CM, Andrieu O, Autard D, Nouaud D, Ashburner M, et al. Combined evidence annotation of transposable elements in genome sequences. PLoS Comp Biol. 2005;1(2):e22. doi: 10.1371/journal.pcbi.0010022 PMC1185648 16110336

[32] Barrón MG, Fiston-Lavier A-S, Petrov DA, González J. Population genomics of transposable elements in Drosophila. Annu Rev Genet. 2014;48(1):561–81. doi: 10.1146/annurev-genet-120213-092359 25292358

[33] Dowsett AP, Young MW. Differing levels of dispersed repetitive DNA among closely related species of Drosophila. Proc Natl Acad Sci U S A. 1982;79(15):4570–4. doi: 10.1073/pnas.79.15.4570 6956880 PMC346716

[34] Rech GE, Radío S, Guirao-Rico S, Aguilera L, Horvath V, Green L, et al. Population-scale long-read sequencing uncovers transposable elements associated with gene expression variation and adaptive signatures in Drosophila. Nat Commun. 2022;13(1):1948. doi: 10.1038/s41467-022-29518-8 35413957 PMC9005704

[35] Deloger M, Cavalli FMG, Lerat E, Biémont C, Sagot M-F, Vieira C. Identification of expressed transposable element insertions in the sequenced genome of Drosophila melanogaster. Gene. 2009;439(1–2):55–62. doi: 10.1016/j.gene.2009.03.015 19332112

[36] Vieira C, Nardon C, Arpin C, Lepetit D, Biémont C. Evolution of genome size in Drosophila. is the invader’s genome being invaded by transposable elements? Mol Biol Evol. 2002;19(7):1154–61. doi: 10.1093/oxfordjournals.molbev.a004173 12082134

[37] Mérel V, Boulesteix M, Fablet M, Vieira C. Transposable elements in Drosophila. Mob DNA. 2020;11:23. doi: 10.1186/s13100-020-00213-z 32636946 PMC7334843

[38] Miller DE, Staber C, Zeitlinger J, Hawley RS. Highly contiguous genome assemblies of 15 Drosophila species generated using nanopore sequencing. G3 (Bethesda). 2018;8(10):3131–41. doi: 10.1534/g3.118.200160 30087105 PMC6169393

[39] Li F, Rane RV, Luria V, Xiong Z, Chen J, Li Z, et al. Phylogenomic analyses of the genus Drosophila reveals genomic signals of climate adaptation. Mol Ecol Resour. 2021;22(4):1559–81. doi: 10.1111/1755-0998.13561 34839580 PMC9299920

[40] Paris M, Boyer R, Jaenichen R, Wolf J, Karageorgi M, Green J, et al. Near-chromosome level genome assembly of the fruit pest Drosophila suzukii using long-read sequencing. Sci Rep. 2020;10(1):11227. doi: 10.1038/s41598-020-67373-z 32641717 PMC7343843

[41] Brncic D, Santibañez SK. The mesophragmatica group of species of drosophila. Evolution. 1957;11(3):300–10. doi: 10.1111/j.1558-5646.1957.tb02899.x

[42] Mota NR, Robe LJ, Valente VLS, Budnik M, Loreto ELS. Phylogeny of the Drosophila mesophragmatica Group (Diptera, Drosophilidae): an example of Andean evolution. Zool Sci. 2008;25(5):526–32. doi: 10.2108/zsj.25.526 18558806

[43] Vela D, Rafael V. Three new andean species of Drosophila (Diptera, Drosophilidae) of the mesophragmatica group. Iheringia Sér Zool. 2004;94(3):295–9. doi: 10.1590/s0073-47212004000300012

[44] Céspedes D, Rafael V. Diversidad del género Drosophila (Diptera, Drosophilidae) en la quebrada de Cruz Loma, Pichincha, Ecuador. REMCB. 2017;34(1–2):215–21. doi: 10.26807/remcb.v34i1-2.245

[45] Pinol J, Francino O, Fontdevila A, Cabré O. Rapid isolation of Drosophila high molecular weight DNA to obtain genomic libraries. Nucl Acids Res. 1988;16(6):2736–2736. doi: 10.1093/nar/16.6.2736 3129700 PMC336417

[46] Andrews S. FastQC a quality control tool for high throughput sequence data. Babraham Bioinformatics [Internet]; 2010 [cited 2023 Aug 12]. Available from: https://www.bioinformatics.babraham.ac.uk/projects/fastqc//

[47] Zimin AV, Marçais G, Puiu D, Roberts M, Salzberg SL, Yorke JA. The MaSuRCA genome assembler. Bioinformatics. 2013;29(21):2669–77. doi: 10.1093/bioinformatics/btt476 23990416 PMC3799473

[48] Trizna M. Assembly_stats. Zenodo; 2020. doi: 10.5281/zenodo.3968774

[49] Simão FA, Waterhouse RM, Ioannidis P, Kriventseva EV, Zdobnov EM. BUSCO: assessing genome assembly and annotation completeness with single-copy orthologs. Bioinformatics. 2015;31(19):3210–2. doi: 10.1093/bioinformatics/btv351 26059717

[50] Pryszcz LP, Gabaldón T. Redundans: an assembly pipeline for highly heterozygous genomes. Nucl Acids Res. 2016;44(12):e113. doi: 10.1093/nar/gkw294 27131372 PMC4937319

[51] Ou S, Su W, Liao Y, Chougule K, Agda JRA, Hellinga AJ, et al. Benchmarking transposable element annotation methods for creation of a streamlined, comprehensive pipeline. Genome Biol. 2019;20(1):275. doi: 10.1186/s13059-019-1905-y 31843001 PMC6913007

[52] Flynn JM, Hubley R, Goubert C, Rosen J, Clark AG, Feschotte C, et al. RepeatModeler2 for automated genomic discovery of transposable element families. Proc Natl Acad Sci U S A. 2020;117(17):9451–7. doi: 10.1073/pnas.1921046117 32300014 PMC7196820

[53] Smith A, Hubley R, Green P. RepeatMasker Open-4.0; 2013–2021.

[54] Riehl K, Riccio C, Miska EA, Hemberg M. TransposonUltimate: software for transposon classification, annotation and detection. Nucl Acids Res. 2022;50(11):e64–e64. doi: 10.1093/nar/gkac136 PMC9226531 35234904

[55] Orozco-Arias S, Sierra P, Durbin R, González J. MCHelper automatically curates transposable element libraries across eukaryotic species. Genome Res. 2024;34(12):2256–68. doi: 10.1101/gr.278821.123 39653419 PMC11694758

[56] Goubert C, Craig RJ, Bilat AF, Peona V, Vogan AA, Protasio AV. A beginner’s guide to manual curation of transposable elements. Mob DNA. 2022;13(1):7. doi: 10.1186/s13100-021-00259-7 35354491 PMC8969392

[57] Tumescheit C, Firth AE, Brown K. CIAlign: a highly customisable command line tool to clean, interpret and visualise multiple sequence alignments. PeerJ. 2022;10:e12983. doi: 10.7717/peerj.12983 35310163 PMC8932311

[58] Kaminker JS, Bergman CM, Kronmiller B, Carlson J, Svirskas R, Patel S, et al. The transposable elements of the Drosophila melanogaster euchromatin: a genomics perspective. Genome Biol. 2002;3(12):RESEARCH0084. doi: 10.1186/gb-2002-3-12-research0084 12537573 PMC151186

[59] Bailly-Bechet M, Haudry A, Lerat E. “One code to find them all”: a perl tool to conveniently parse RepeatMasker output files. Mobile DNA. 2014;5(1):1–15. doi: 10.1186/1759-8753-5-1324382139

[60] Neph S, Kuehn MS, Reynolds AP, Haugen E, Thurman RE, Johnson AK, et al. BEDOPS: high-performance genomic feature operations. Bioinformatics. 2012;28(14):1919–20. doi: 10.1093/bioinformatics/bts277 22576172 PMC3389768

[61] Quinlan AR, Hall IM. BEDTools: a flexible suite of utilities for comparing genomic features. Bioinformatics. 2010;26(6):841–2. doi: 10.1093/bioinformatics/btq033 20110278 PMC2832824

[62] Benson G. Tandem repeats finder: a program to analyze DNA sequences. Nucl Acids Res. 1999;27(2):573–80. doi: 10.1093/nar/27.2.573 9862982 PMC148217

[63] Novák P, Neumann P, Pech J, Steinhaisl J, Macas J. RepeatExplorer: a Galaxy-based web server for genome-wide characterization of eukaryotic repetitive elements from next-generation sequence reads. Bioinformatics. 2013;29(6):792–3. doi: 10.1093/bioinformatics/btt054 23376349

[64] Afgan E, Baker D, Batut B, van den Beek M, Bouvier D, Cech M, et al. The Galaxy platform for accessible, reproducible and collaborative biomedical analyses: 2018 update. Nucl Acids Res. 2018;46(W1):W537–44. doi: 10.1093/nar/gky379 29790989 PMC6030816

[65] Camacho C, Coulouris G, Avagyan V, Ma N, Papadopoulos J, Bealer K, et al. BLAST+: architecture and applications. BMC Bioinform. 2009;10:421. doi: 10.1186/1471-2105-10-421 20003500 PMC2803857

[66] de Lima LG, Ruiz-Ruano FJ. In-depth satellitome analyses of 37 Drosophila species illuminate repetitive DNA evolution in the Drosophila genus. Genome Biol Evol. 2022;14(5):evac064. doi: 10.1093/gbe/evac064 35511582 PMC9113345

[67] Silva BSML, Picorelli ACR, Kuhn GCS. In silico identification and characterization of satellite DNAs in 23 Drosophila species from the Montium Group. Genes (Basel). 2023;14(2):300. doi: 10.3390/genes14020300 36833227 PMC9957191

[68] Ruiz-Ruano FJ, López-León MD, Cabrero J, Camacho JPM. High-throughput analysis of the satellitome illuminates satellite DNA evolution. Sci Rep. 2016;6:28333. doi: 10.1038/srep28333 27385065 PMC4935994

[69] Craddock EM, Gall JG, Jonas M. Hawaiian Drosophila genomes: size variation and evolutionary expansions. Genetica. 2016;144(1):107–24. doi: 10.1007/s10709-016-9882-5 26790663

[70] Gregory TR. Animal genome size database (release 2.0). In: Animal genome size database (release 2.0); 2015.

[71] Drosophila 12 Genomes Consortium, Clark AG, Eisen MB, Smith DR, Bergman CM, Oliver B, et al. Evolution of genes and genomes on the Drosophila phylogeny. Nature. 2007;450(7167):203–18. doi: 10.1038/nature06341 17994087

[72] Lerat E. Identifying repeats and transposable elements in sequenced genomes: how to find your way through the dense forest of programs. Heredity (Edinb). 2010;104(6):520–33. doi: 10.1038/hdy.2009.165 19935826

[73] Fonseca PM, Moura RD, Wallau GL, Loreto ELS. The mobilome of Drosophila incompta, a flower-breeding species: comparison of transposable element landscapes among generalist and specialist flies. Chromosome Res. 2019;27(3):203–19. doi: 10.1007/s10577-019-09609-x 31119502

[74] Storer JM, Hubley R, Rosen J, Smit AFA. Methodologies for the De novo Discovery of Transposable Element Families. Genes (Basel). 2022;13(4):709. doi: 10.3390/genes13040709 35456515 PMC9025800

[75] Loreto EL, da Silva LB, Zaha A, Valente VL. Distribution of transposable elements in neotropical species of Drosophila. Genetica. 1997;101(3):153–65. doi: 10.1023/a:1018381104700 9692225

[76] Germanos E, Mota NR, Loreto ELS. Transposable elements from the mesophragmatica group of Drosophila. Genet Mol Biol. 2006;29(4):741–6. doi: 10.1590/s1415-47572006000400026

[77] Silva JC, Kidwell MG. Horizontal transfer and selection in the evolution of P elements. Mol Biol Evol. 2000;17(10):1542–57. doi: 10.1093/oxfordjournals.molbev.a026253 11018160

[78] Loreto EL, Valente VL, Zaha A, Silva JC, Kidwell MG. Drosophila mediopunctata P elements: a new example of horizontal transfer. J Hered. 2001;92(5):375–81. doi: 10.1093/jhered/92.5.375 11773243

[79] Liu K, Wessler SR. Functional characterization of the active Mutator-like transposable element, Muta1 from the mosquito Aedes aegypti. Mob DNA. 2017;8:1. doi: 10.1186/s13100-016-0084-6 28096902 PMC5225508

[80] Tan S, Ma H, Wang J, Wang M, Wang M, Yin H, et al. DNA transposons mediate duplications via transposition-independent and -dependent mechanisms in metazoans. Nat Commun. 2021;12(1):4280. doi: 10.1038/s41467-021-24585-9 34257290 PMC8277862

[81] Dupeyron M, Singh KS, Bass C, Hayward A. Evolution of Mutator transposable elements across eukaryotic diversity. Mobile DNA. 2019;10(1). doi: 10.1186/s13100-019-0153-8 PMC6446971 30988700

[82] Tang Z, Zhang H-H, Huang K, Zhang X-G, Han M-J, Zhang Z. Repeated horizontal transfers of four DNA transposons in invertebrates and bats. Mob DNA. 2015;6(1):3. doi: 10.1186/s13100-014-0033-1 25606061 PMC4298943

[83] Fedoroff NV. Molecular genetics and epigenetics of CACTA elements. Methods Mol Biol. 2013;1057:177–92. doi: 10.1007/978-1-62703-568-2_13 23918429

[84] Jacobson JW, Medhora MM, Hartl DL. Molecular structure of a somatically unstable transposable element in Drosophila. Proc Natl Acad Sci U S A. 1986;83(22):8684–8. doi: 10.1073/pnas.83.22.8684 3022302 PMC386995

[85] Palazzo A, Moschetti R, Caizzi R, Marsano RM. The Drosophila mojavensis Bari3 transposon: distribution and functional characterization. Mob DNA. 2014;5:21. doi: 10.1186/1759-8753-5-21 25093043 PMC4120734

[86] Palazzo A, Lorusso P, Miskey C, Walisko O, Gerbino A, Marobbio CMT, et al. Transcriptionally promiscuous “blurry” promoters in Tc1/mariner transposons allow transcription in distantly related genomes. Mob DNA. 2019;10:13. doi: 10.1186/s13100-019-0155-6 30988701 PMC6446368

[87] Palazzo A, Caizzi R, Moschetti R, Marsano RM. What have we learned in 30 years of investigations on bari transposons? Cells. 2022;11(3):583. doi: 10.3390/cells11030583 35159391 PMC8834629

[88] Bargues N, Lerat E. Evolutionary history of LTR-retrotransposons among 20 Drosophila species. Mob DNA. 2017;8:7. doi: 10.1186/s13100-017-0090-3 28465726 PMC5408442

[89] Flavell AJ, Pearce SR, Heslop-Harrison P, Kumar A. The evolution of Ty1-copia group retrotransposons in eukaryote genomes. Genetica. 1997;100(1–3):185–95. doi: 10.1023/a:1018385713293 9440272

[90] Rubin PM, Loreto ELS, Carareto CMA, Valente VLS. The copia retrotransposon and horizontal transfer in Drosophila willistoni. Genet Res (Camb). 2011;93(3):175–80. doi: 10.1017/S0016672310000625 21450134

[91] Distribution and conservation of mobile elements in the genus Drosophila. Mol Biol Evol. 1986;3:522–34. doi: 10.1093/oxfordjournals.molbev.a040413 2832695

[92] Santos R de CO dos, Vilela CR. Breeding sites of Neotropical Drosophilidae (Diptera): IV. living and fallen flowers of Sessea brasiliensis and Cestrum spp. (Solanaceae). Rev Bras Entomol. 2005;49(4):544–51. doi: 10.1590/s0085-56262005000400015

[93] Rius N, Guillén Y, Delprat A, Kapusta A, Feschotte C, Ruiz A. Exploration of the Drosophila buzzatii transposable element content suggests underestimation of repeats in Drosophila genomes. BMC Genomics. 2016;17:344. doi: 10.1186/s12864-016-2648-8 27164953 PMC4862133

[94] Sicat JPA, Visendi P, Sewe SO, Bouvaine S, Seal SE. Characterization of transposable elements within the Bemisia tabaci species complex. Mob DNA. 2022;13(1):12. doi: 10.1186/s13100-022-00270-6 35440097 PMC9017028

[95] Deprá M, Ludwig A, Valente VL, Loreto EL. Mar, a MITE family of hAT transposons in Drosophila. Mob DNA. 2012;3(1):13. doi: 10.1186/1759-8753-3-13 22935191 PMC3517528

[96] Bergman CM, Quesneville H, Anxolabéhère D, Ashburner M. Recurrent insertion and duplication generate networks of transposable element sequences in the Drosophila melanogaster genome. Genome Biol. 2006;7(11):R112. doi: 10.1186/gb-2006-7-11-r112 17134480 PMC1794594

[97] Bologa AM, Stoica I, Constantin ND, Ecovoiu AAl. The landscape of the DNA transposons in the genome of the Horezu_LaPeri strain of Drosophila melanogaster. Insects. 2023;14(6):494. doi: 10.3390/insects14060494 37367310 PMC10299278

[98] Bergman CM, Bensasson D. Recent LTR retrotransposon insertion contrasts with waves of non-LTR insertion since speciation in Drosophila melanogaster. Proc Natl Acad Sci U S A. 2007;104(27):11340–5. doi: 10.1073/pnas.0702552104 17592135 PMC2040900

[99] Kofler R, Nolte V, Schlötterer C. Tempo and mode of transposable element activity in Drosophila. PLoS Genet. 2015;11(7):e1005406. doi: 10.1371/journal.pgen.1005406 PMC4505896 26186437

[100] Gregory TR, Johnston JS. Genome size diversity in the family Drosophilidae. Heredity. 2008;101(3):228–38. doi: 10.1038/hdy.2008.49 18523443

[101] Moriyama EN, Petrov DA, Hartl DL. Genome size and intron size in Drosophila. Mol Biol Evol. 1998;15(6):770–3. doi: 10.1093/oxfordjournals.molbev.a025980 9615458

[102] Schlötterer C, Harr B. Drosophila virilis has long and highly polymorphic microsatellites. Mol Biol Evol. 2000;17(11):1641–6. doi: 10.1093/oxfordjournals.molbev.a026263 11070052

[103] Shah A, Hoffman JI, Schielzeth H. Comparative analysis of genomic repeat content in gomphocerine grasshoppers reveals expansion of satellite DNA and helitrons in species with unusually large genomes. Genome Biol Evol. 2020;12(7):1180–93. doi: 10.1093/gbe/evaa119 32539114 PMC7486953

[104] Lohe AR, Brutlag DL. Identical satellite DNA sequences in sibling species of Drosophila. J Mol Biol. 1987;194(2):161–70. doi: 10.1016/0022-2836(87)90365-2 3112413

[105] Montiel EE, Mora P, Rico-Porras JM, Palomeque T, Lorite P. Satellitome of the Red Palm Weevil, Rhynchophorus ferrugineus (Coleoptera: Curculionidae), the most diverse among insects. Front Ecol Evol. 2022;10:826808. doi: 10.3389/fevo.2022.826808

[106] Oppert B, Dossey AT, Chu F-C, Šatović-Vukšić E, Plohl M, Smith TPL, et al. The genome of the yellow mealworm, Tenebrio molitor: it’s bigger than you think. Genes. 2023;14(12):2209. doi: 10.3390/genes14122209 38137031 PMC10742464

[107] Mora P, Rico-Porras JM, Palomeque T, Montiel EE, Pita S, Cabral-de-Mello DC, et al. Satellitome analysis of Adalia bipunctata (Coleoptera): revealing centromeric turnover and potential chromosome rearrangements in a comparative interspecific study. IJMS. 2024;25(17):9214. doi: 10.3390/ijms25179214 39273162 PMC11394905

[108] Gržan T, Dombi M, Despot-Slade E, Veseljak D, Volarić M, Meštrović N, et al. The low-copy-number satellite DNAs of the model beetle Tribolium castaneum. Genes (Basel). 2023;14(5):999. doi: 10.3390/genes14050999 37239359 PMC10218199

[109] Majid M, Yuan H. Comparative analysis of transposable elements in genus Calliptamus grasshoppers revealed that satellite DNA contributes to genome size variation. Insects. 2021;12(9):837. doi: 10.3390/insects12090837 34564277 PMC8466570

[110] Kuhn GCS. “Satellite DNA transcripts have diverse biological roles in Drosophila”. Heredity (Edinb). 2015;115(1):1–2. doi: 10.1038/hdy.2015.12 25806543 PMC4815497

[111] Rico-Porras JM, Mora P, Palomeque T, Montiel EE, Cabral-de-Mello DC, Lorite P. Heterochromatin is not the only place for satDNAs: the high diversity of satDNAs in the euchromatin of the beetle Chrysolina americana (Coleoptera, Chrysomelidae). Genes (Basel). 2024;15(4):395. doi: 10.3390/genes15040395 38674330 PMC11049206

[112] Anjos A, Milani D, Bardella VB, Paladini A, Cabral-de-Mello DC. Evolution of satDNAs on holocentric chromosomes: insights from hemipteran insects of the genus Mahanarva. Chromosome Res. 2023;31(1):1–15. doi: 10.1007/s10577-023-09710-2 36705735

[113] Melters DP, Bradnam KR, Young HA, Telis N, May MR, Ruby JG, et al. Comparative analysis of tandem repeats from hundreds of species reveals unique insights into centromere evolution. Genome Biol. 2013;14(1):R10. doi: 10.1186/gb-2013-14-1-r10 23363705 PMC4053949

[114] Kuhn GCS, Heringer P, Dias GB. Structure, organization, and evolution of satellite DNAs: insights from the Drosophila repleta and D. virilis species groups. Prog Mol Subcell Biol. 2021;60:27–56. doi: 10.1007/978-3-030-74889-0_2 34386871

[115] Palomeque T, Lorite P. Satellite DNA in insects: a review. Heredity (Edinb). 2008;100(6):564–73. doi: 10.1038/hdy.2008.24 18414505

[116] Palacios-Gimenez OM, Koelman J, Palmada-Flores M, Bradford TM, Jones KK, Cooper SJB, et al. Comparative analysis of morabine grasshopper genomes reveals highly abundant transposable elements and rapidly proliferating satellite DNA repeats. BMC Biol. 2020;18(1):199. doi: 10.1186/s12915-020-00925-x 33349252 PMC7754599

[117] Mora P, Vela J, Ruiz-Ruano FJ, Ruiz-Mena A, Montiel EE, Palomeque T, et al. Satellitome analysis in the ladybird beetle Hippodamia variegata (Coleoptera, Coccinellidae). Genes. 2020;11(7):783. doi: 10.3390/genes11070783 32668664 PMC7397073

[118] Félix AP, Amorim IC de, Milani D, Cabral-de-Mello DC, Moura RC. Differential amplification and contraction of satellite DNAs in the distinct lineages of the beetle Euchroma gigantea. Gene. 2024;927:148723. doi: 10.1016/j.gene.2024.148723 38914242

[119] Garrido-Ramos MA. Satellite DNA: an evolving topic. Genes (Basel). 2017;8(9):230. doi: 10.3390/genes8090230 28926993 PMC5615363

[120] Hill T. Transposable element dynamics are consistent across the Drosophila phylogeny, despite drastically differing content. bioRxiv. 2019:651059. doi: 10.1101/651059

[121] Gebert D, Hay AD, Hoang JP, Gibbon AE, Henderson IR, Teixeira FK. Analysis of 30 chromosome-level Drosophila genome assemblies reveals dynamic evolution of centromeric satellite repeats. Genome Biol. 2025;26(1):63. doi: 10.1186/s13059-025-03527-4 40102968 PMC11917152

[122] Canapa A, Barucca M, Biscotti MA, Forconi M, Olmo E. Transposons, genome size, and evolutionary insights in animals. Cytogenet Genome Res. 2015;147(4):217–39. doi: 10.1159/000444429 26967166

[123] Nardon C, Weiss M, Vieira C, Biémont C. Variation of the genome size estimate with environmental conditions in Drosophila melanogaster. Cytometry A. 2003;55(1):43–9. doi: 10.1002/cyto.a.10061 12938187

[124] Bartolomé C, Charlesworth B. Rates and patterns of chromosomal evolution in Drosophila pseudoobscura and D. miranda. Genetics. 2006;173(2):779–91. doi: 10.1534/genetics.105.054585 16547107 PMC1526542

[125] Yasuhara J, Wakimoto B. Oxymoron no more: the expanding world of heterochromatic genes. TIG. 2006;22(6):330–8. doi: 10.1016/j.tig.2006.04.008 16690158

[126] Levine MT, Jones CD, Kern AD, Lindfors HA, Begun DJ. Novel genes derived from noncoding DNA in Drosophila melanogaster are frequently X-linked and exhibit testis-biased expression. Proc Natl Acad Sci USA. 2006;103(26):9935–9. doi: 10.1073/pnas.0509809103 16777968 PMC1502557

[127] Casola C, Lawing AM, Betrán E, Feschotte C. PIF-like transposons are common in drosophila and have been repeatedly domesticated to generate new host genes. Mol Biol Evol. 2007;24(8):1872–88. doi: 10.1093/molbev/msm116 17556756

[128] Heckenhauer J, Frandsen PB, Sproul JS, Li Z, Paule J, Larracuente AM, et al. Genome size evolution in the diverse insect order Trichoptera. Gigascience. 2022;11:giac011. doi: 10.1093/gigascience/giac011 35217860 PMC8881205

[129] Bao R, Friedrich M. Genomic signatures of globally enhanced gene duplicate accumulation in the megadiverse higher Diptera fueling intralocus sexual conflict resolution. PeerJ. 2020;8:e10012. doi: 10.7717/peerj.10012 33083121 PMC7560327

[130] Bao R, Dia SE, Issa HA, Alhusein D, Friedrich M. Comparative evidence of an exceptional impact of gene duplication on the developmental evolution of Drosophila and the higher diptera. Front Ecol Evol. 2018;6:322427. doi: 10.3389/fevo.2018.00063

[131] Julca I, Marcet-Houben M, Cruz F, Vargas-Chavez C, Johnston JS, Gómez-Garrido J, et al. Phylogenomics identifies an ancestral burst of gene duplications predating the diversification of Aphidomorpha. Mol Biol Evol. 2020;37(3):730–56. doi: 10.1093/molbev/msz261 31702774 PMC7038657

[132] Rossato DO, Ludwig A, Deprá M, Loreto ELS, Ruiz A, Valente VLS. BuT2 is a member of the third major group of hAT transposons and is involved in horizontal transfer events in the genus Drosophila. Genome Biol Evol. 2014;6(2):352–65. doi: 10.1093/gbe/evu017 24459285 PMC3942097

[133] Guio L, González J. New insights on the evolution of genome content: population dynamics of transposable elements in flies and humans. Methods Mol Biol. 2019;1910:505–30. doi: 10.1007/978-1-4939-9074-0_16 31278675

[134] Sessegolo C, Burlet N, Haudry A. Strong phylogenetic inertia on genome size and transposable element content among 26 species of flies. Biol Lett. 2016;12(8):20160407. doi: 10.1098/rsbl.2016.0407 27576524 PMC5014035

[135] Sproul JS, Hotaling S, Heckenhauer J, Powell A, Marshall D, Larracuente AM, et al. Analyses of 600+ insect genomes reveal repetitive element dynamics and highlight biodiversity-scale repeat annotation challenges. Genome Res. 2023;33(10):1708–17. doi: 10.1101/gr.277387.122 37739812 PMC10691545

