# Correction: Identification of transposable elements and satellite DNA in the Neotropical species *Drosophila amaguana* from the Ecuadorian Andean Forests

**DOI:** 10.1371/journal.pone.0344113

**Published:** 2026-03-03

**Authors:** Manuel Alejandro Coba-Males, Simon Orozco-Arias, Romain Guyot, Doris Vela

The authors, Manuel Alejandro Coba-Males, Romain Guyot and Doris Vela, should be noted as shared co-corresponding authors on this work. Romain Guyot’s email address is: romain.guyot@ird.fr.
