# Supplementary material for: Identification of transposable elements and satellite DNA in the Neotropical species Drosophila amaguana from the Ecuadorian Andean Forests
Source: PLoS One. 2025 Dec 10;20(12):e0337390. doi: 10.1371/journal.pone.0337390 (PMC12694884; doi:10.1371/journal.pone.0337390)
Supplement: S1 File — (PDF) [file pone.0337390.s001.pdf]

FastQC Report

dom 18 ago 2024
Damag_1.fastq.gz

Summary

- Basic Statistics
- Per base sequence quality
- Per tile sequence quality
- Per sequence quality scores
- Per base sequence content
- Per sequence GC content
- Per base N content
- Sequence Length Distribution
- Sequence Duplication Levels
- Overrepresented sequences
- Adapter Content

Basic Statistics

Measure	Value
Filename	Damag_1.fastq.gz
File type	Conventional base calls
Encoding	Sanger / Illumina 1.9
Total Sequences	143613112
Total Bases	21.6 Gbp
Sequences flagged as poor quality	0
Sequence length	151
%GC	44

Per base sequence quality

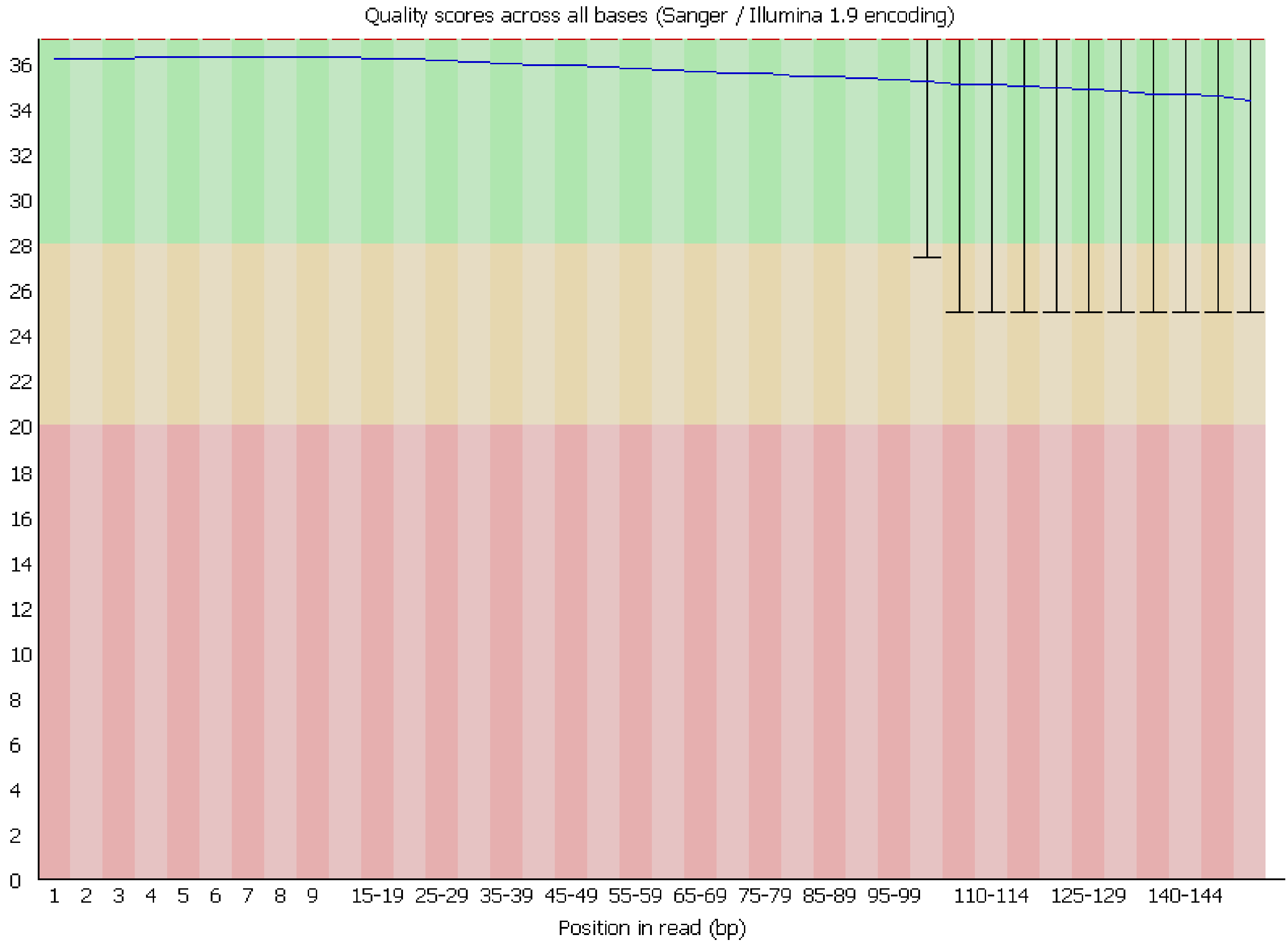

Per tile sequence quality

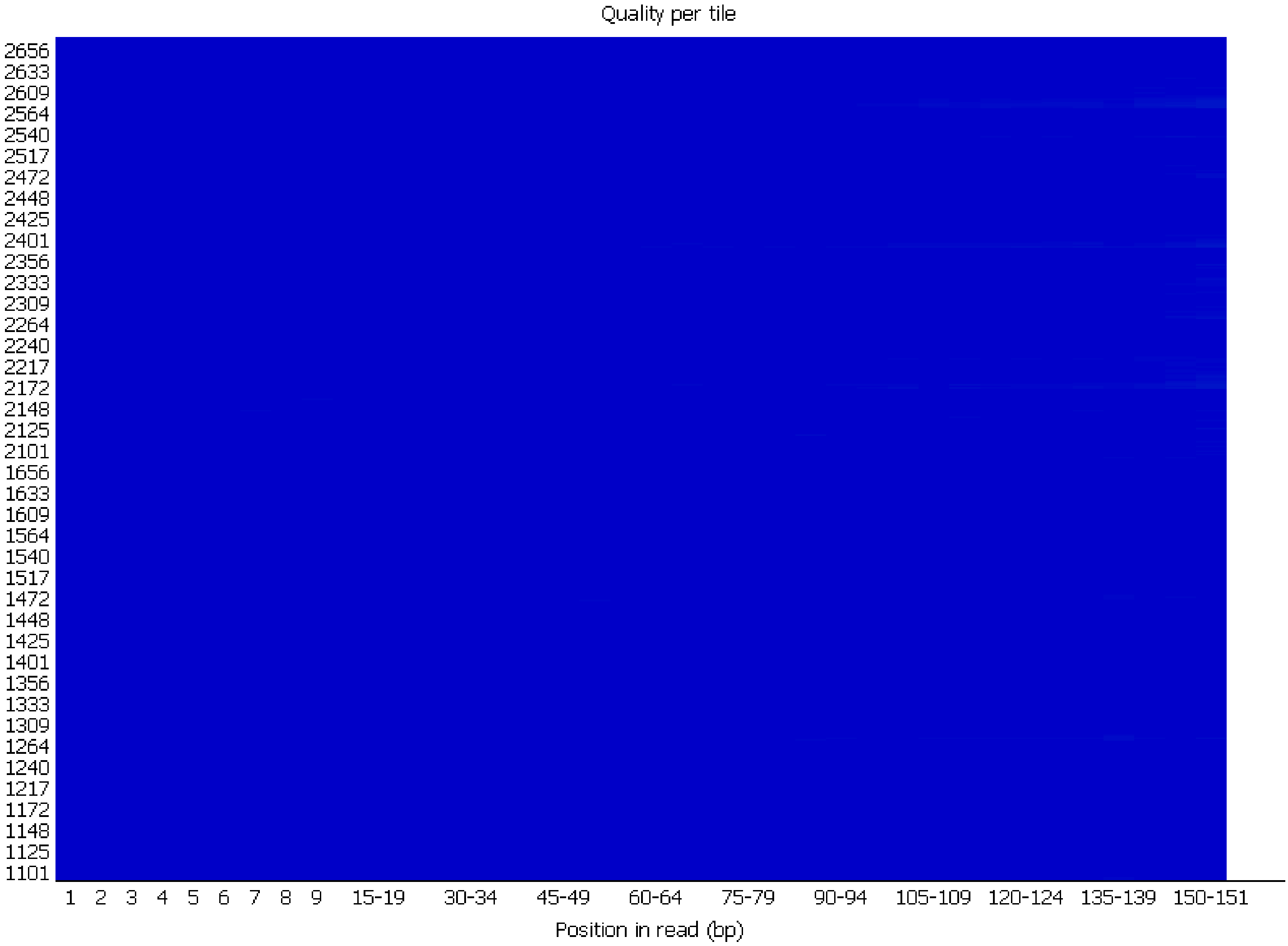

Per sequence quality scores

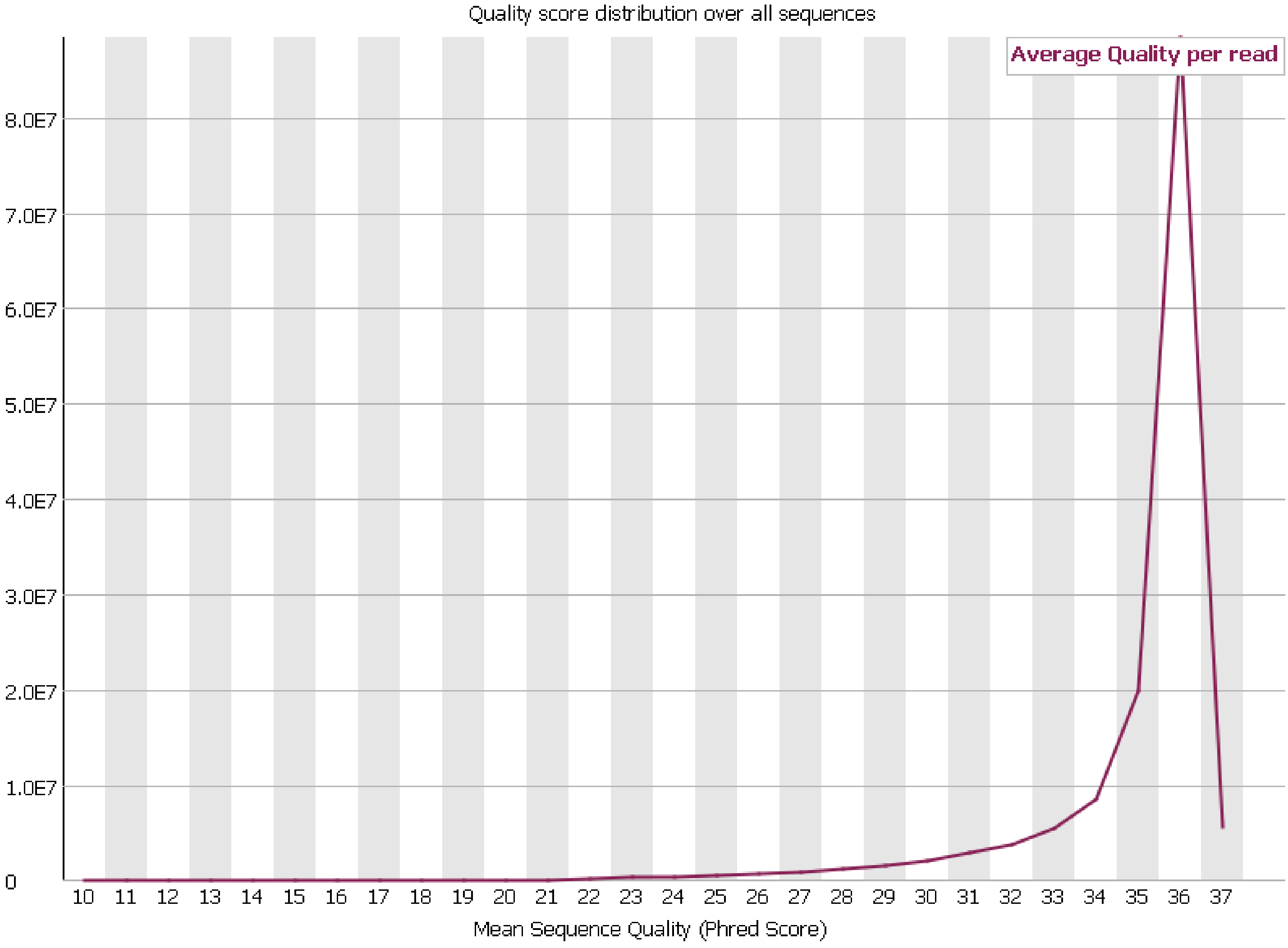

Per base sequence content

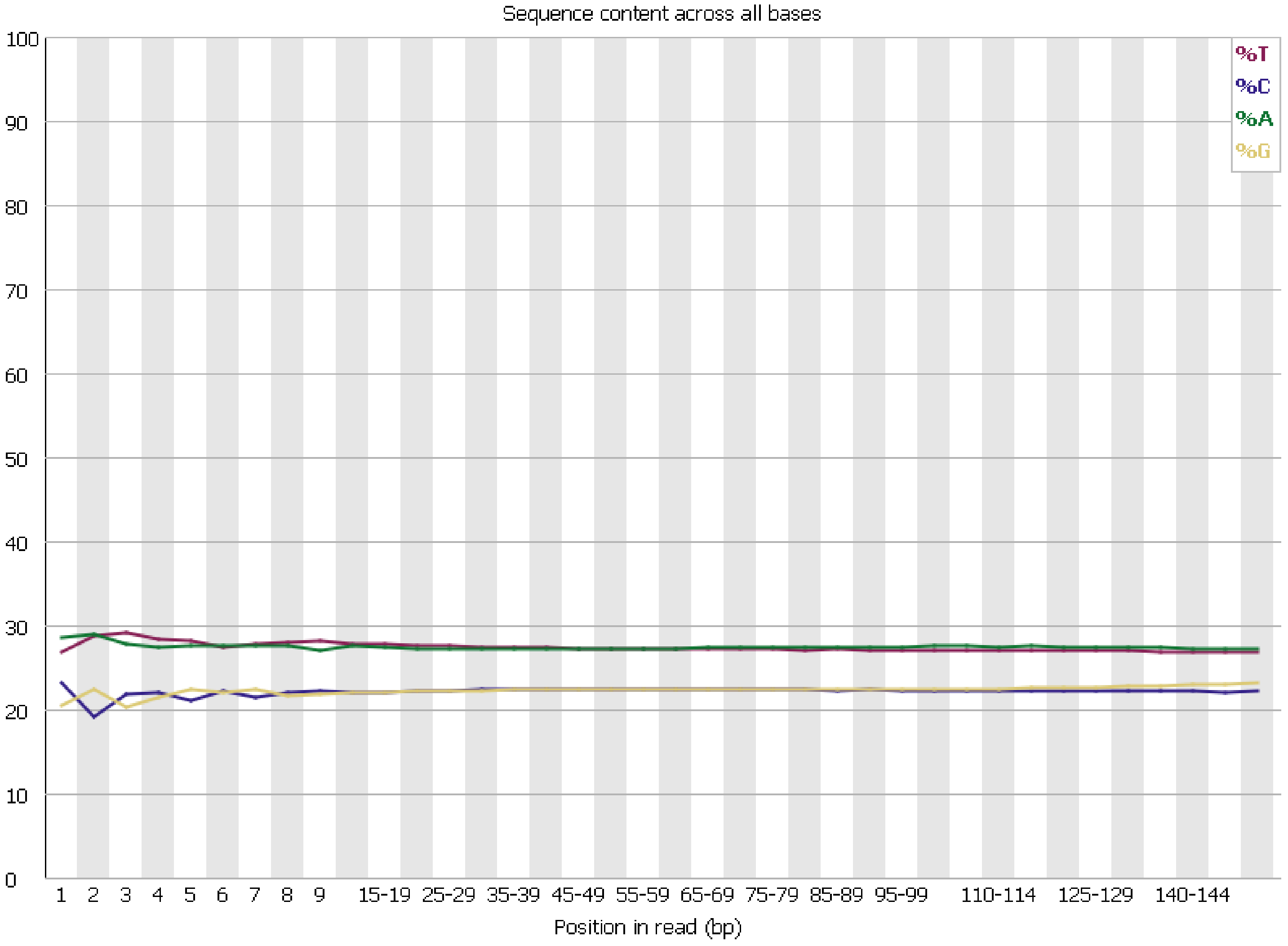

Per sequence GC content

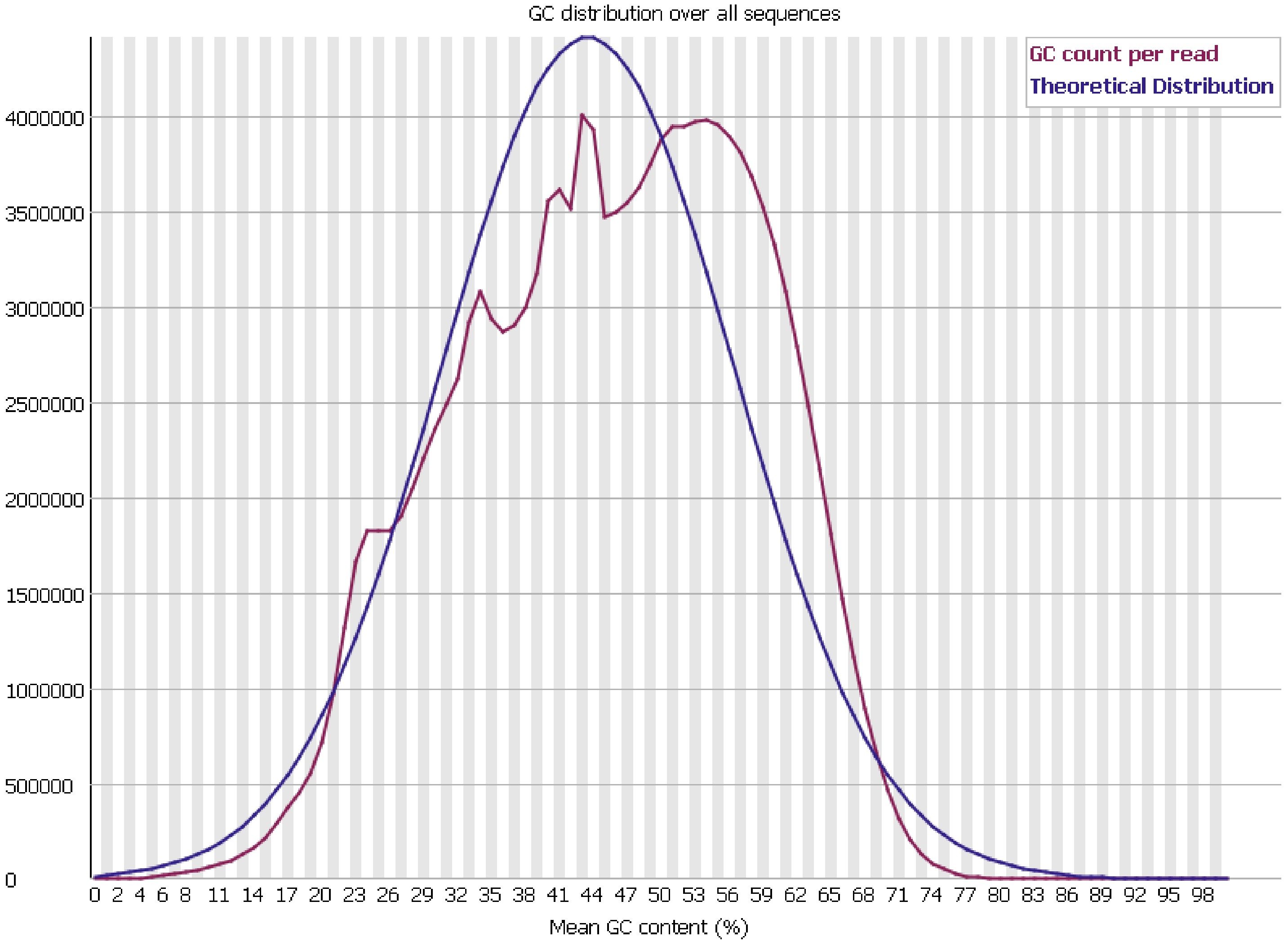

Per base N content

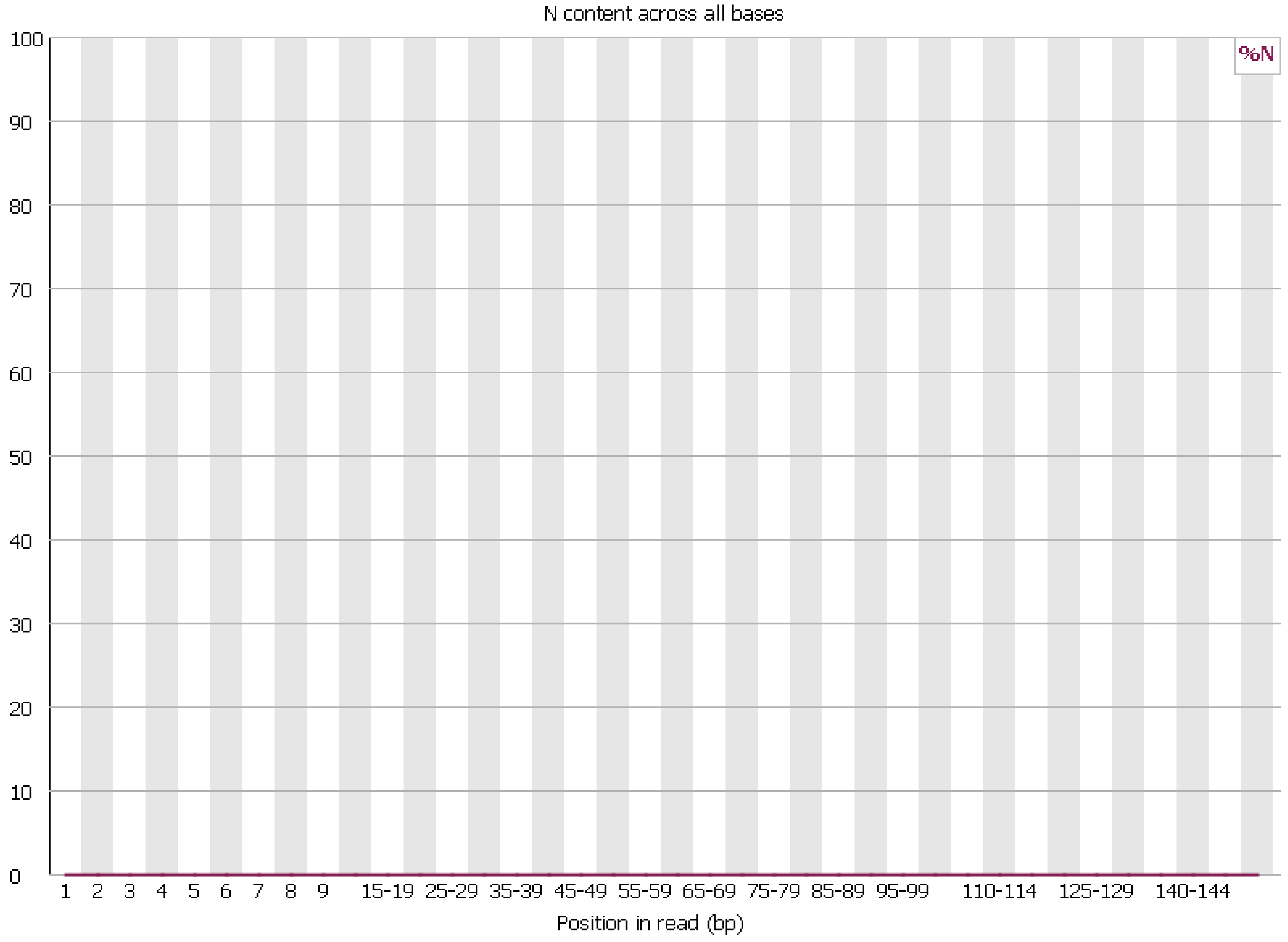

Sequence Length Distribution

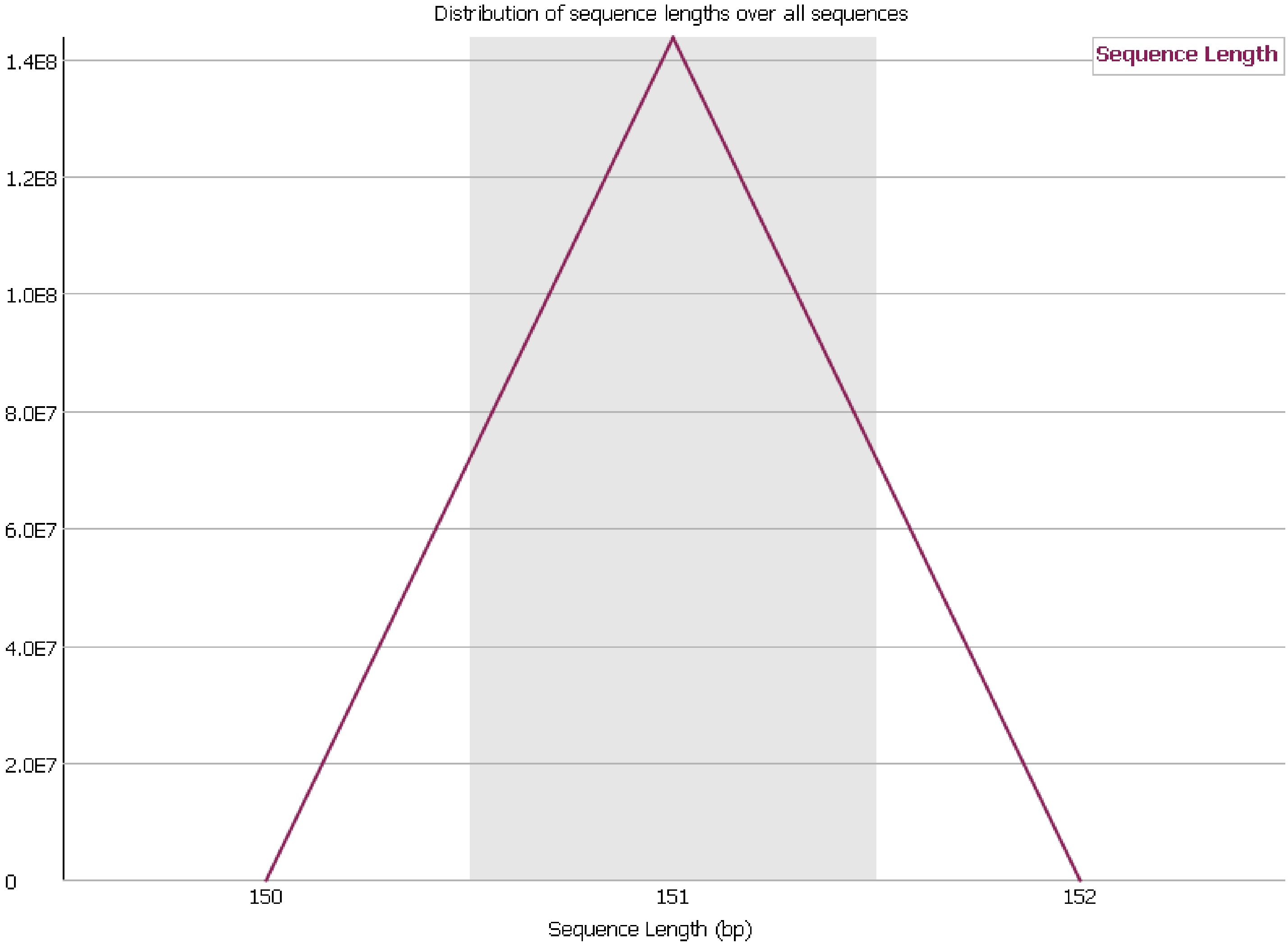

Sequence Duplication Levels

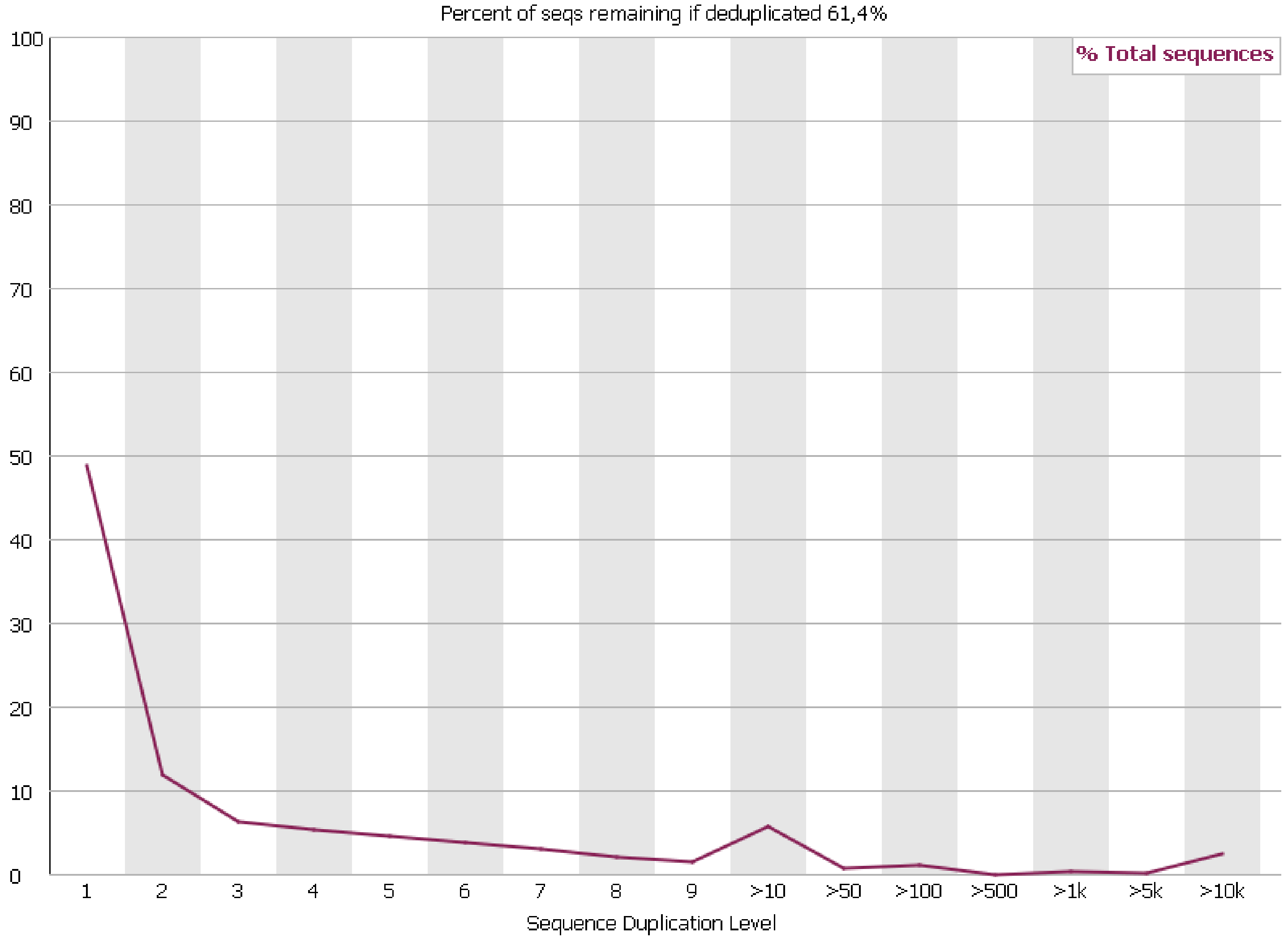

Overrepresented sequences

No overrepresented sequences

Adapter Content

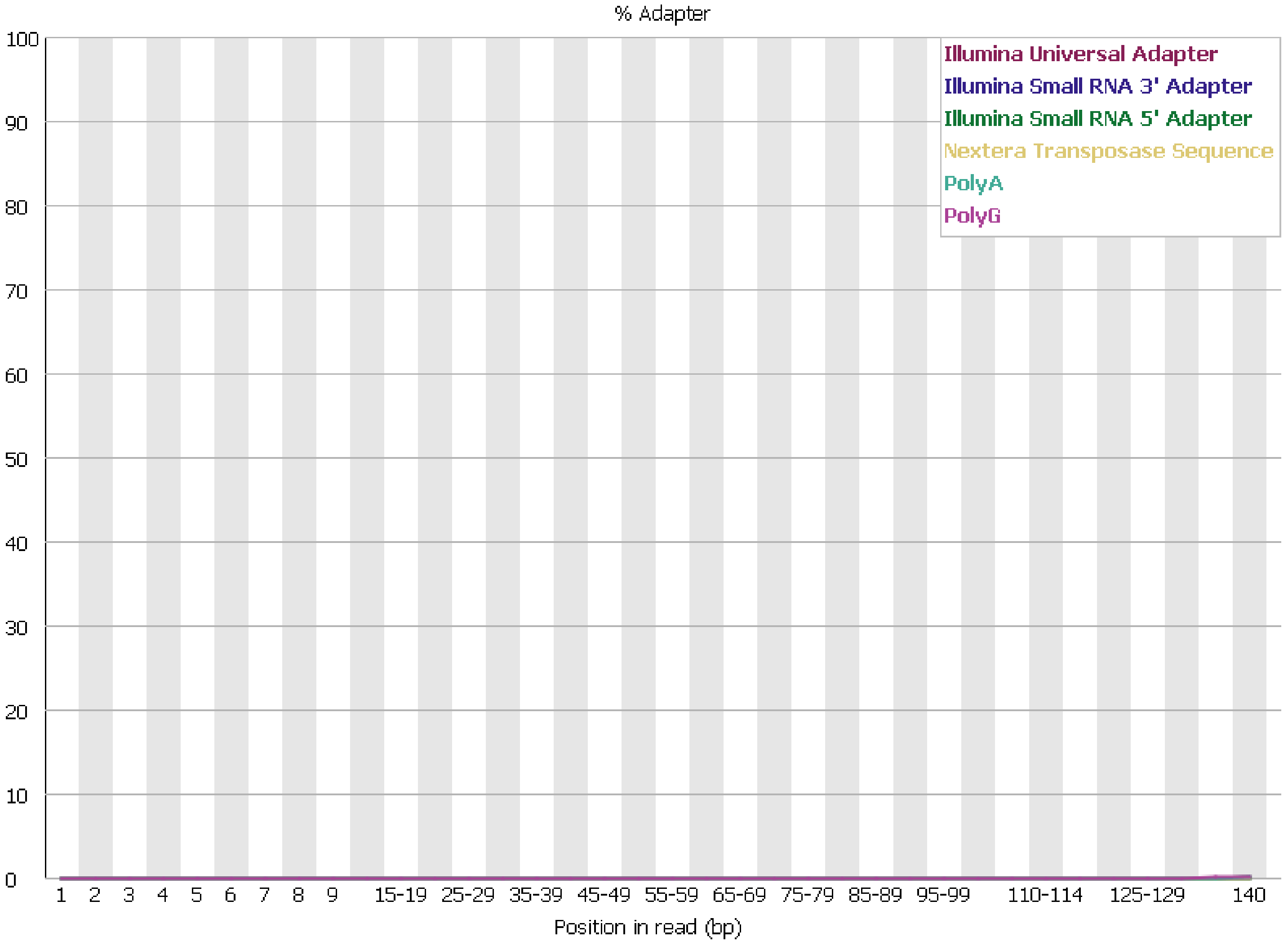

Produced by FastQC (version 0.12.1)

FastQC Report

dom 18 ago 2024
Damag_2.fastq.gz

Summary

- Basic Statistics
- Per base sequence quality
- Per tile sequence quality
- Per sequence quality scores
- Per base sequence content
- Per sequence GC content
- Per base N content
- Sequence Length Distribution
- Sequence Duplication Levels
- Overrepresented sequences
- Adapter Content

Basic Statistics

Measure	Value
Filename	Damag_2.fastq.gz
File type	Conventional base calls
Encoding	Sanger / Illumina 1.9
Total Sequences	143613112
Total Bases	21.6 Gbp
Sequences flagged as poor quality	0
Sequence length	151
%GC	45

Per base sequence quality

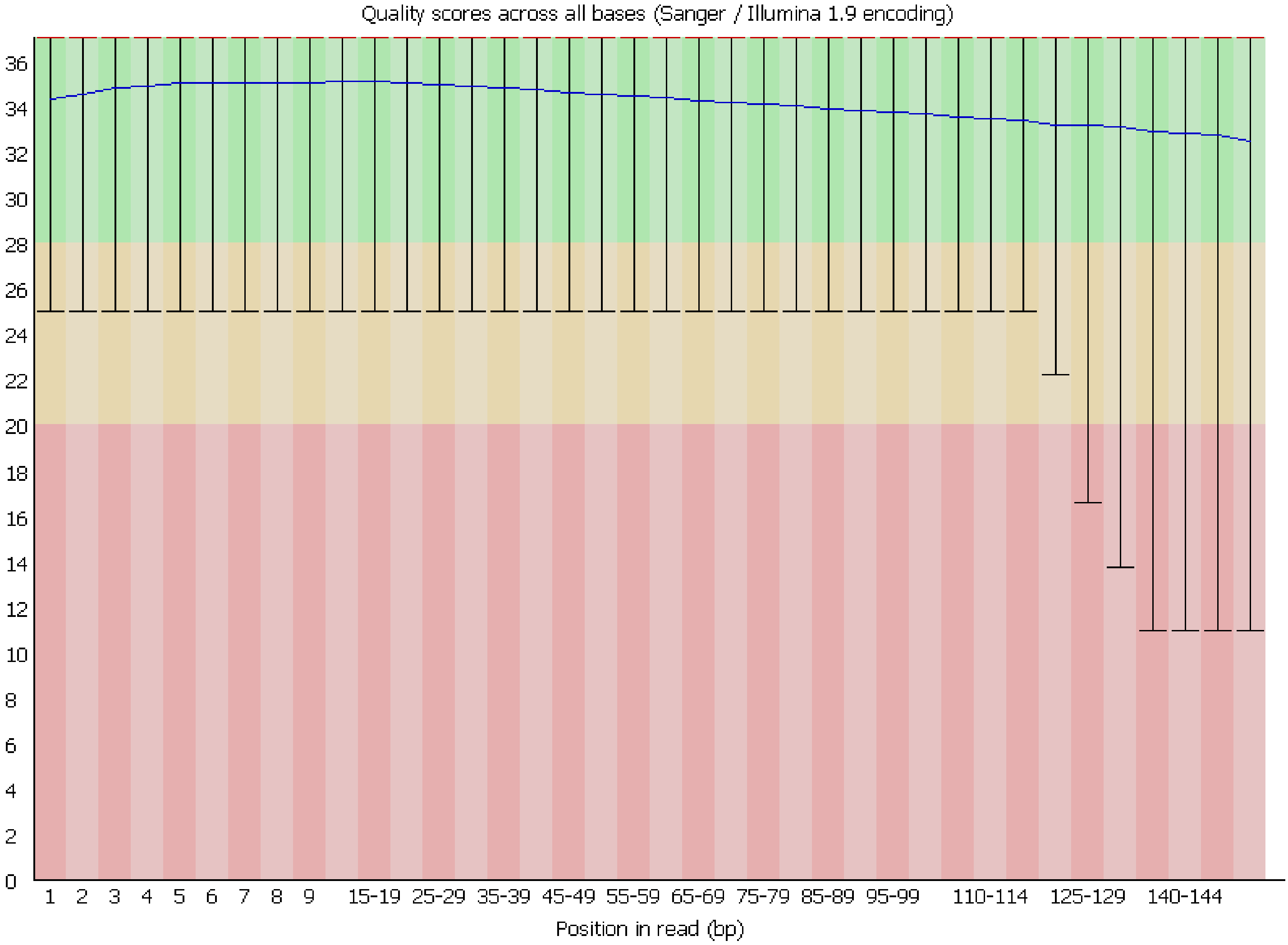

Per tile sequence quality

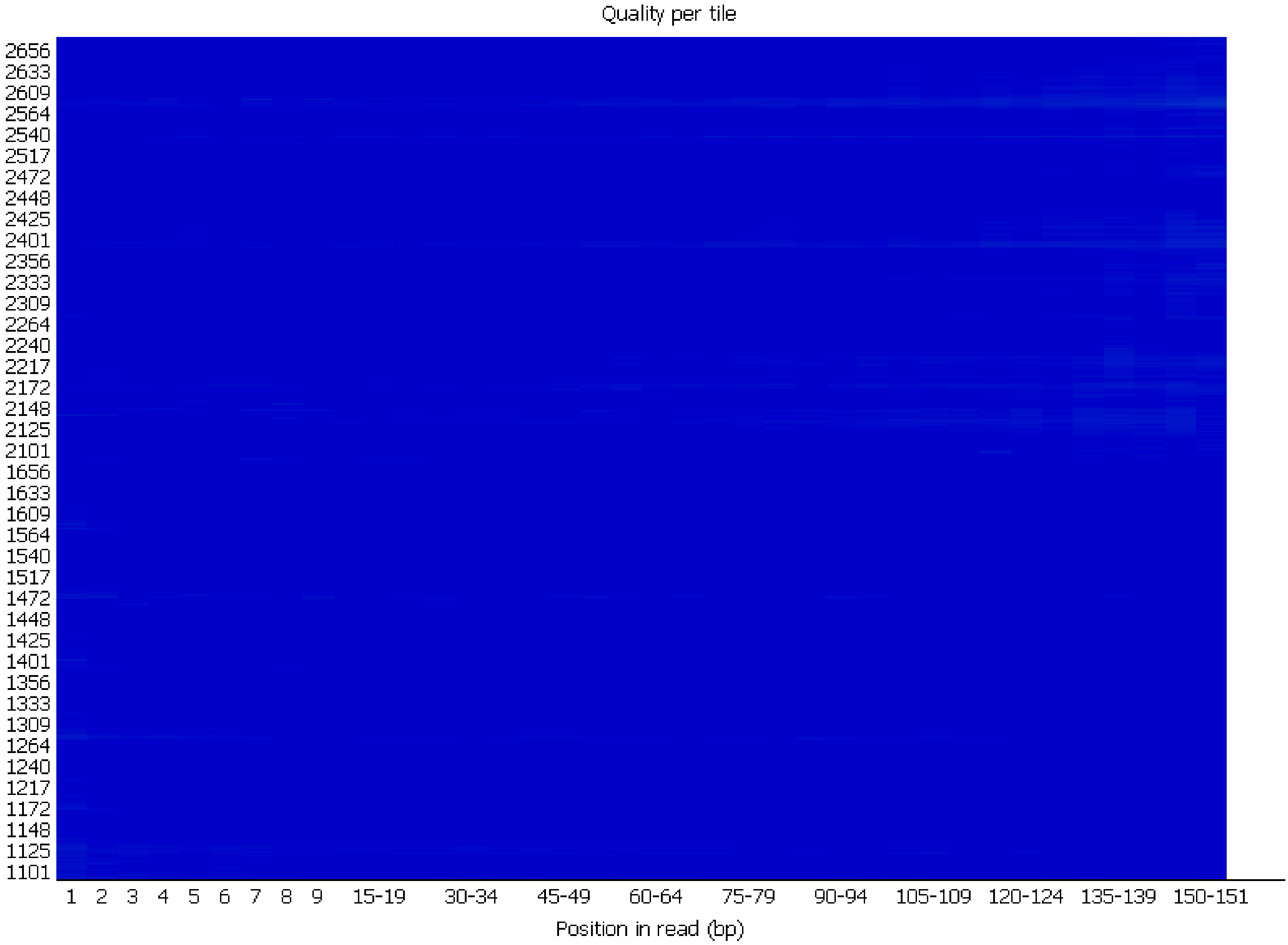

Per sequence quality scores

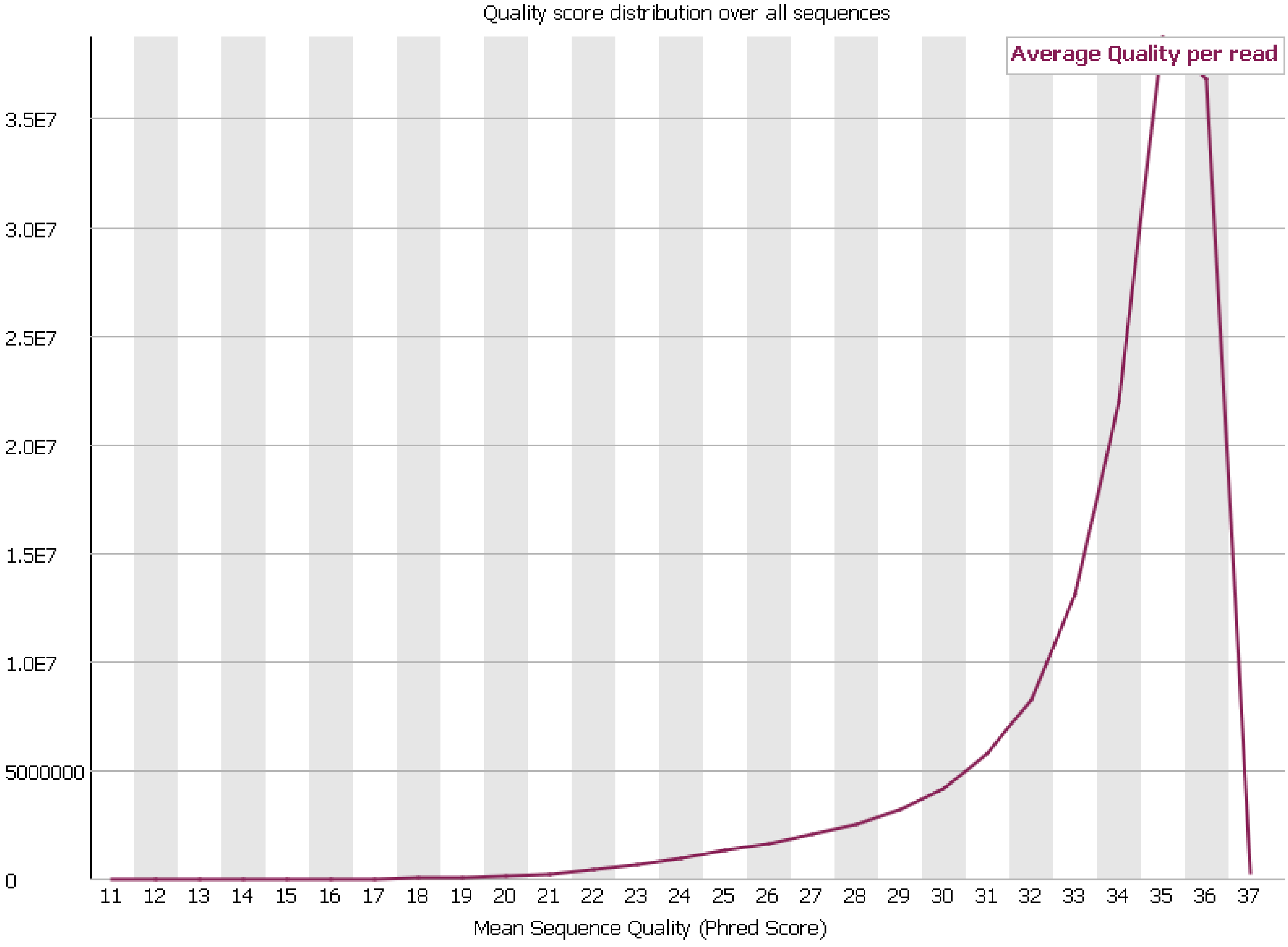

Per base sequence content

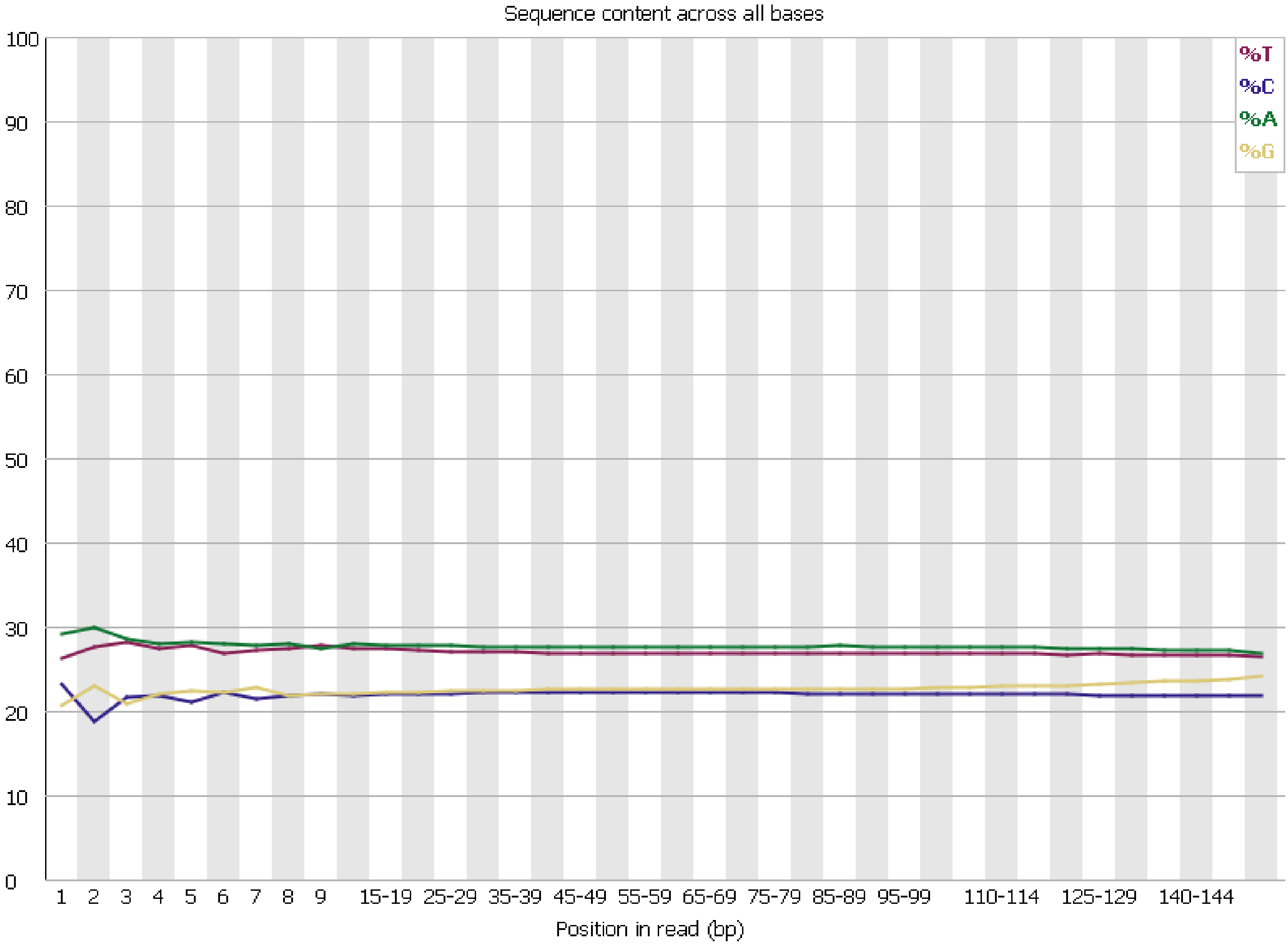

Per sequence GC content

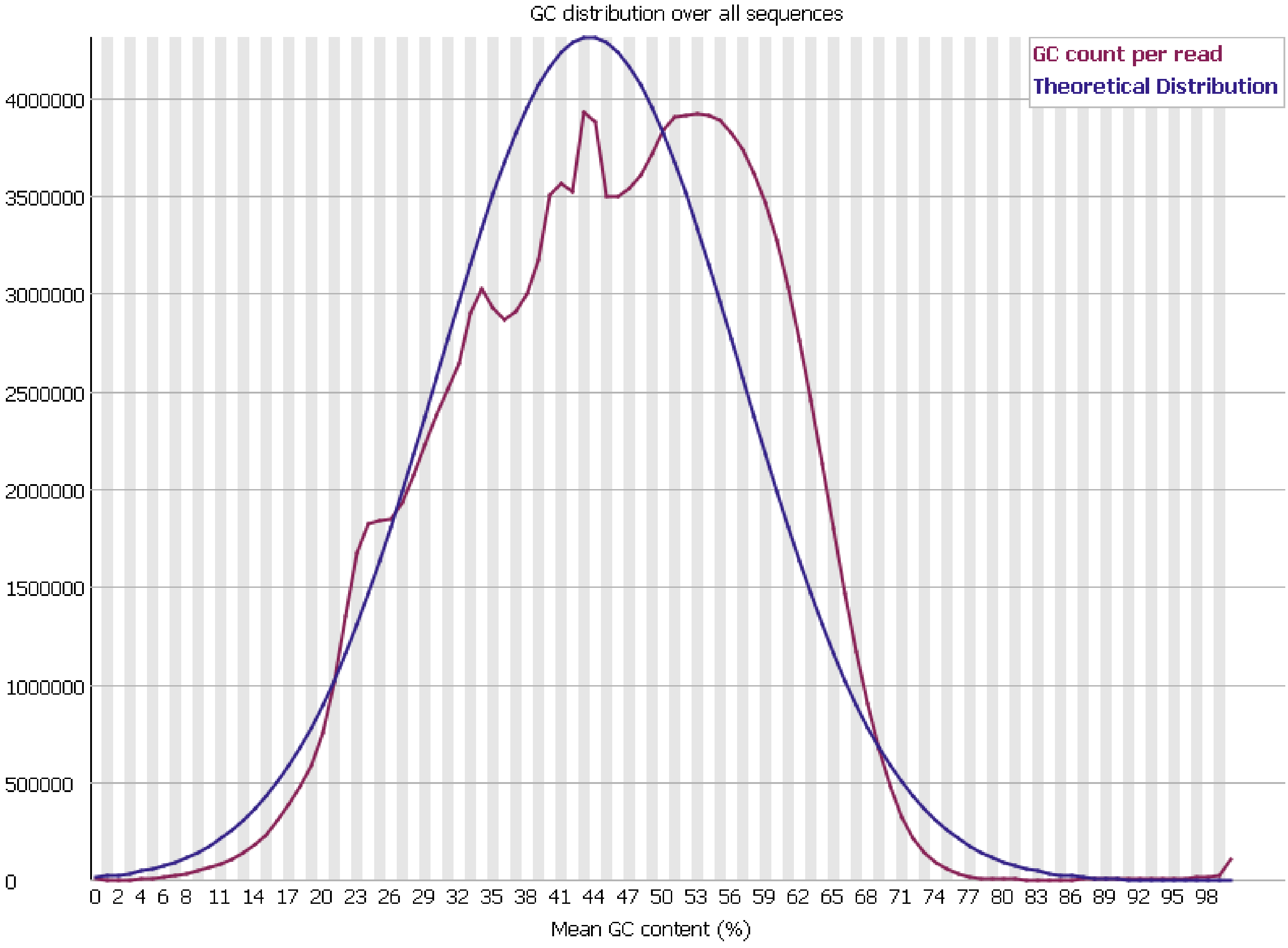

Per base N content

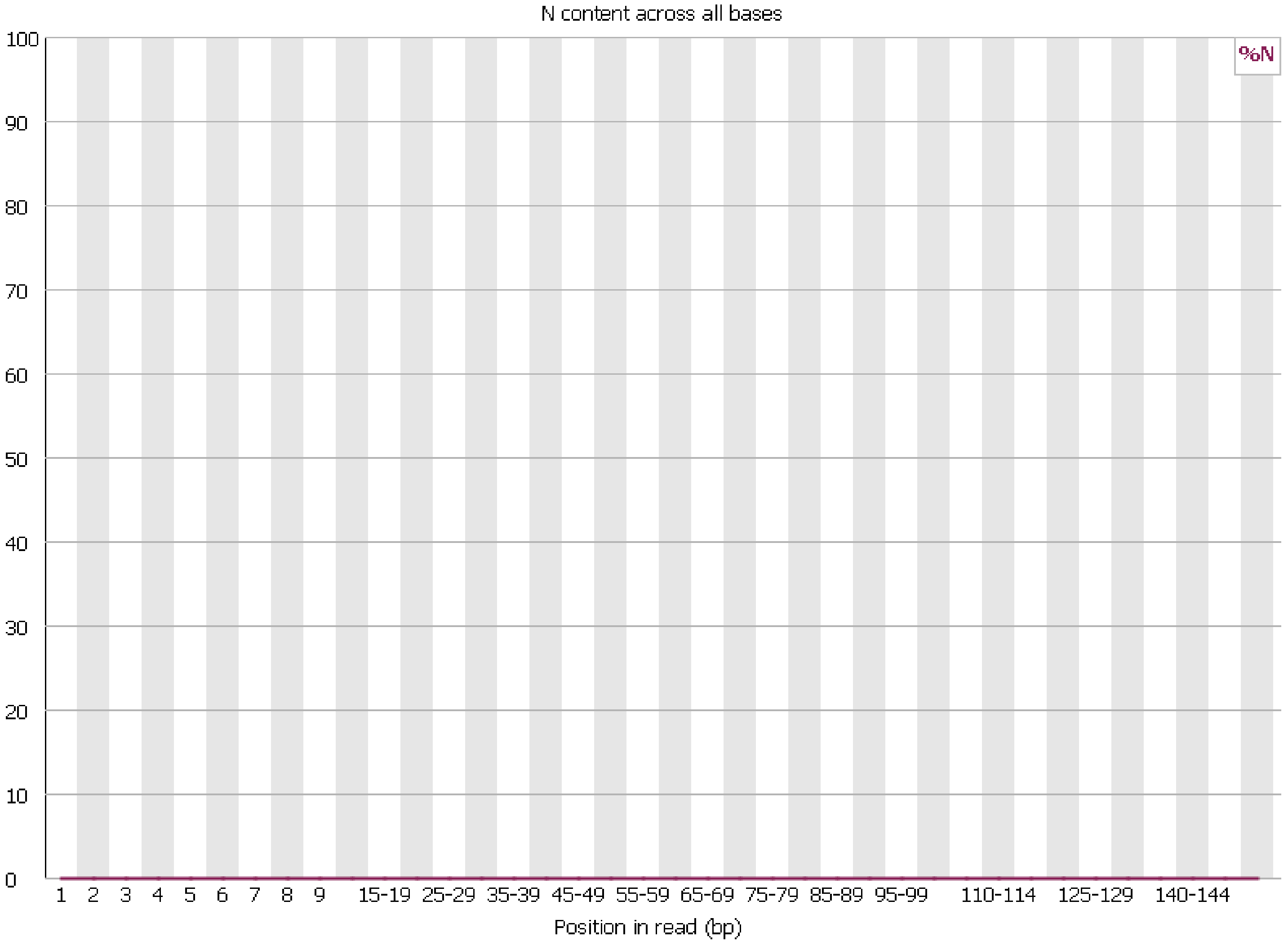

Sequence Length Distribution

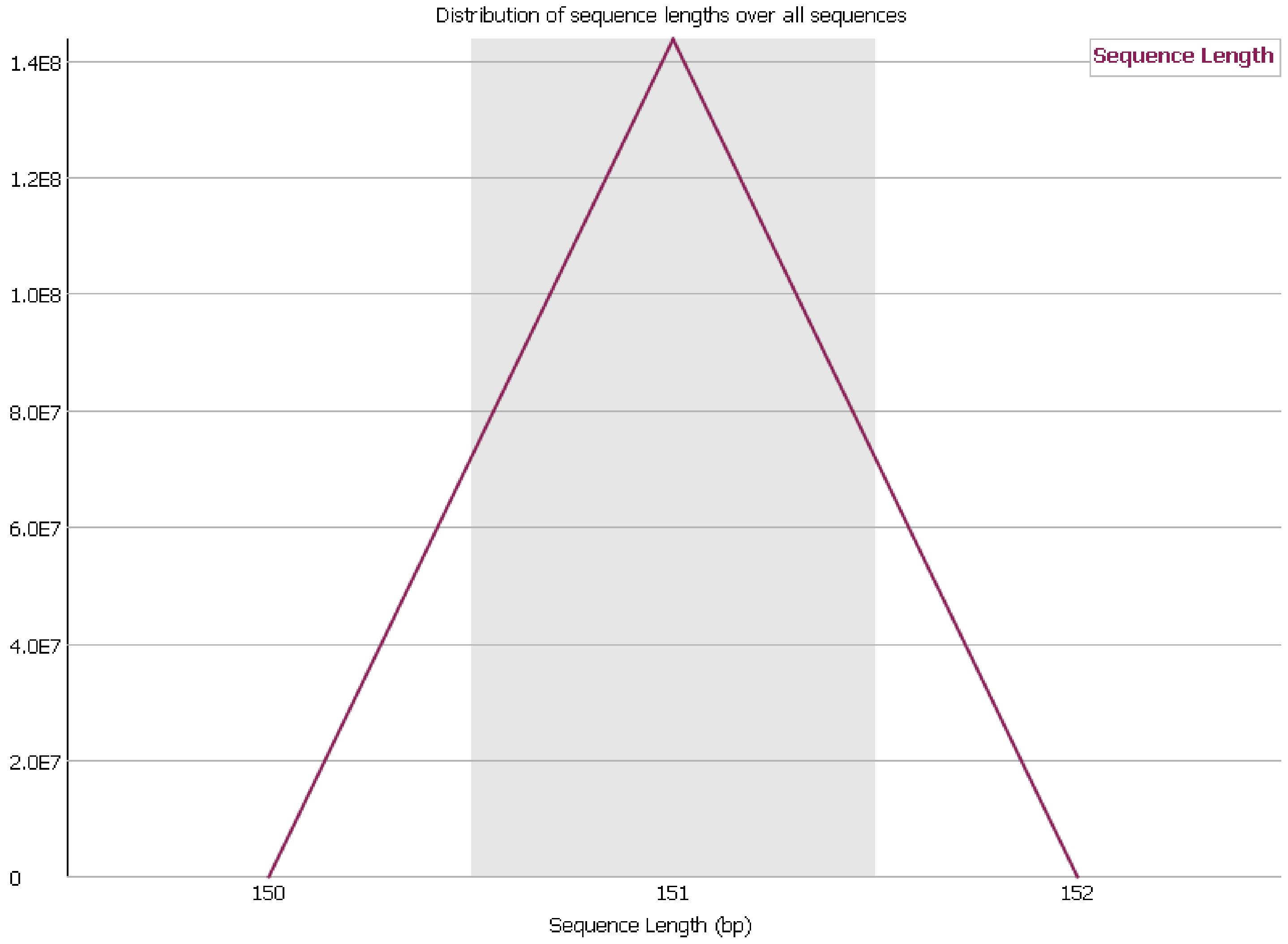

Sequence Duplication Levels

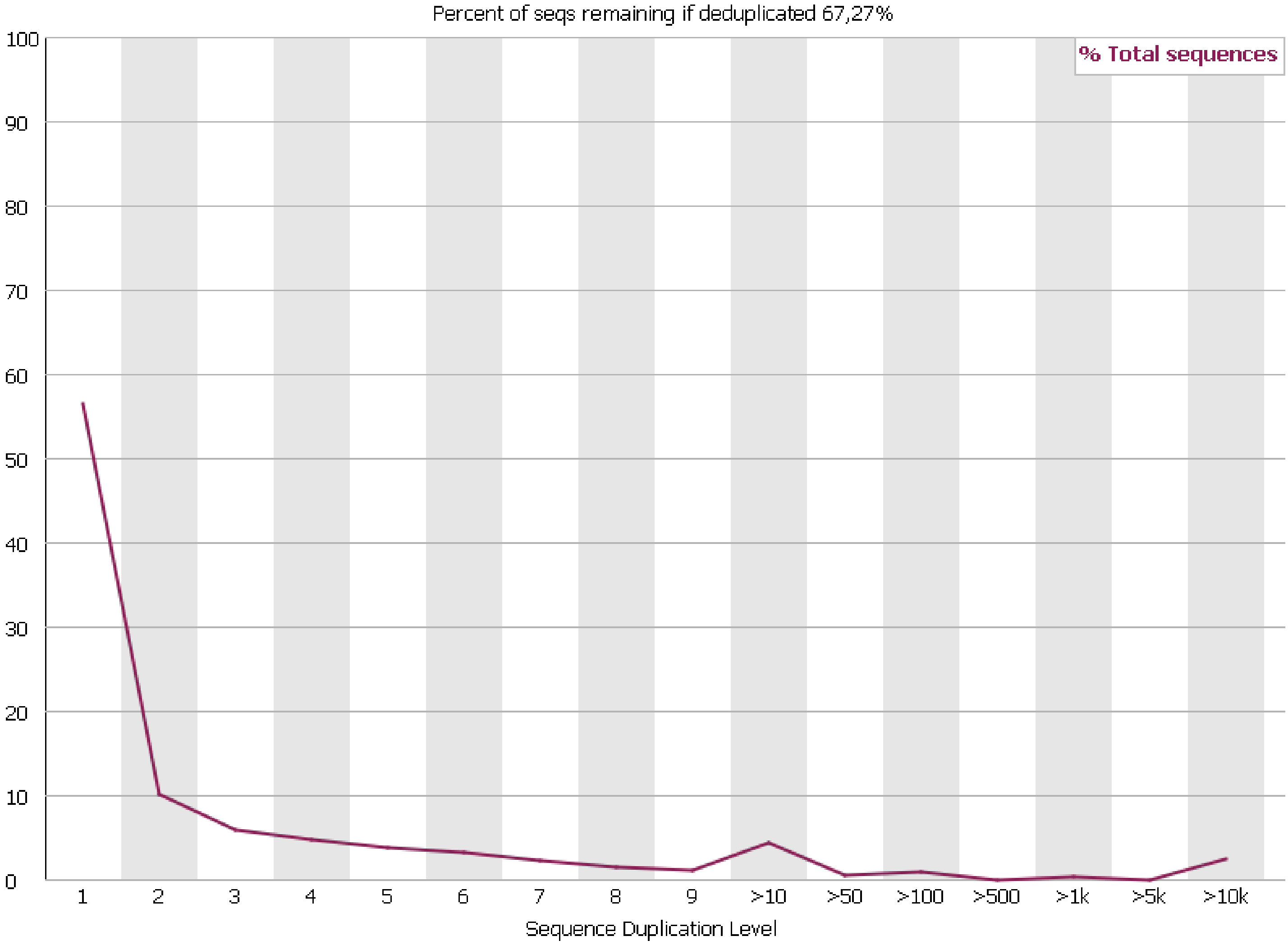

Overrepresented sequences

Sequence	Count	Percentage	Possible Source
GGGGGGGGGGGGGGGGGGGGGGGGGGGGGGGGGGGGGGGGGGGGGGGGGG	240128	0.16720478837614772	No Hit

Adapter Content

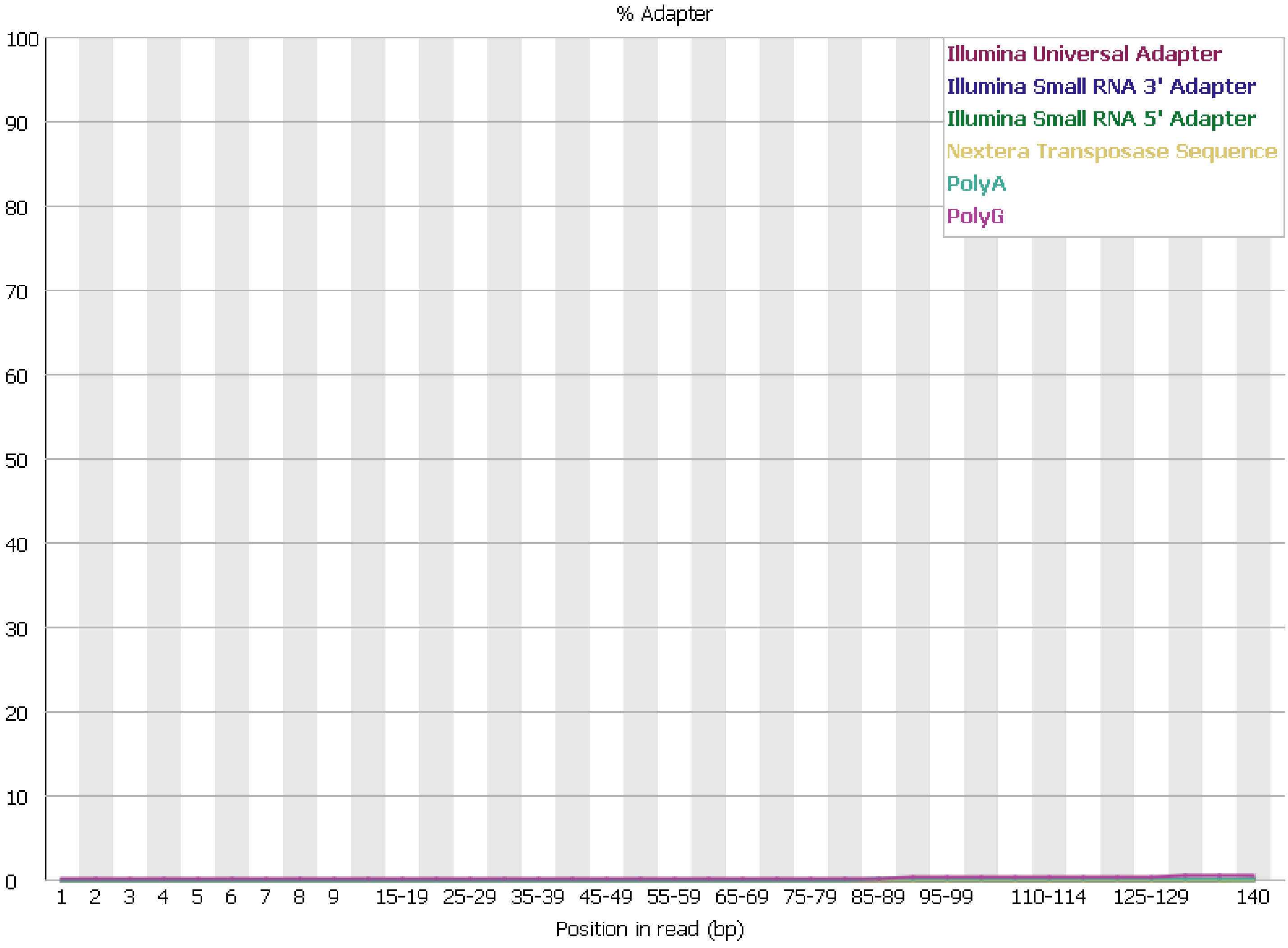

Produced by FastQC (version 0.12.1)
