# Supplementary material for: Identification of transposable elements and satellite DNA in the Neotropical species Drosophila amaguana from the Ecuadorian Andean Forests
Source: PLoS One. 2025 Dec 10;20(12):e0337390. doi: 10.1371/journal.pone.0337390 (PMC12694884; doi:10.1371/journal.pone.0337390)
Supplement: S2 File — (PDF) [file pone.0337390.s002.pdf]

Additional File 2: Supplementary Tables

Table S1. Summary statistics of the *D. amaguana* genome assembly before and after redundancy reduction using Redundans.

**Genome before Redundans steps**	***D. amaguana***
**Genome size[a] (bp)**	455,546,830
***N* Contigs**	75,476
**Contigs N_{50} (kb)**	28.854
**Longest contig (Mb)**	2.415
**Heterozygous size (bp)**	151,236
**Heterozygous size (%)**	0.03
***N* Heterozygous contigs**	1,004
**Heterozygous contigs (%)**	1.33
**Identity (%)**	0.000
**Possible joins**	0
**Homozygous size (bp)**	455,395,594
**Homozygous size[b] (%)**	99.97
***N* Homozygous contigs**	74,472
**Homozygous contigs (%)**	98.67
**Genome after Redundans steps**	***D. amaguana***
**Genome size[b] (bp)**	455,790,300
***N* Contigs**	69,679
**Contig N_{50} (kb)**	29.533
**Longest contig (Mb)**	2.415

The table summarizes key assembly statistics of the draft genome assembly of *D. amaguana* genome before and after redundancy reduction using Redundans, applied to the initial draft assembly generated with MaSuRCA v.4.0.0. Metrics include genome size, contig counts, contig N_{50}, longest contig, and heterozygous and homozygous contig sizes (in base pairs and percentages). Redundans was used primarily to validate that the original genome size was not overestimated due to duplicated or heterozygous regions. The unchanged genome size after scaffolding supports the accuracy of the initial draft assembly length and provides insights into genome contiguity, heterozygosity, and overall assembly quality. [a]Initial genome size in the draft assembly of *D. amaguana* from MaSuRCA. [b]Percentage of the initial draft assembly identified as homozygous before Redundans analysis. [c]Genome size of the draft assembly removing duplicated regions using Redundans.

Table S2. Consensus sequences in the manually curated TE library for *D. amaguana* complying with the Wicker rule (80-80-80), showing correspondence with transposable elements (TEs) previously described in other species.

TE order	TE superfamily	Organism	TE name[a]
LTR	RLX	*D. melanogaster*	con13_UnFmcl013_RLX[b]
	Gypsy	*D. willistoni*	Gypsy-5_DWil-I
	Gypsy	*D. willistoni*	Gypsy-5_DWil-I_2
	Gypsy	*D. willistoni*	Gypsy-5_DWil-I_3
	Gypsy	*D. willistoni*	Gypsy-5_DWil-I_4
	Gypsy	*D. willistoni*	Gypsy-5_DWil-I_5
	Gypsy	*D. willistoni*	Gypsy-5_DWil-I_6
	Gypsy	*D. willistoni*	Gypsy-5_DWil-I_7
	Gypsy	*D. willistoni*	Gypsy-45_DWil-I
	Gypsy	*D. ananassae*	Gypsy-38_DAn-I
	Gypsy	*D. ananassae*	Gypsy-38_DAn-I_2
LINE	R1	*D. mercatorum*	FBte0001036
	RTE	*D. bipectinata*	RTE-2_DBp
	RTE	*D. bipectinata*	RTE-2_DBp_2
	Jockey	*D. persimilis*	Jockey-5_DPer
	Jockey	*D. persimilis*	Jockey-5_DPer_2
TIR	Tc1-Mariner	*D. elegans*	Mariner-1_DEl
	Tc1-Mariner	*L. salmonis*	Mariner-1_LSal
	Tc1-Mariner	*D. hydei*	FBte0000026
Helitron	Helitron	*D. virilis*	Helitron-1_DVir
	Helitron	*D. virilis*	Helitron-1_Dvir_2

[a]The sequences for these transposable elements names are available from the Berkeley Drosophila Genome Project (BDGP) dataset (https://www.fruitfly.org/) (Kaminker et al., 2002)[1] and the Manual Curated TE library (MCTE) (Rech et al., 2022)[2]. [b]Sequence labeled as incomplete by MCHelper, as it did not pass the structural check in fully automatic mode during manual curation of the TE library.

[1] Kaminker, J., Bergman, C., Kronmiller, B., Carlson, J., Svirskas, R., Patel, S., Frise, E., Wheeler, D., Lewis, S., Rubin, G., Ashburner, M., & Celniker, S. (2002). The transposable elements of the Drosophila melanogaster: euchromatin a genomics perspective. *Genome Biology*, *3*(12), 1–20. https://doi.org/10.1186/gb-2002-3-12-research0084

[2] Rech, G. E., Radío, S., Guirao-Rico, S., Aguilera, L., Horvath, V., Green, L., Lindstadt, H., Jamilloux, V., Quesneville, H., & González, J. (2022). Population-scale long-read sequencing uncovers transposable elements associated with gene expression variation and adaptive signatures in Drosophila. *Nature Communications 2022 13:1*, *13*(1), 1–16. https://doi.org/10.1038/s41467-022-29518-8

Table S3. General overview of transposable element (TE) annotation in the *D. amaguana* genome by order, including number of copies, genome proportion (%), and total length (bp).

TE order	No. Copies	Copies (%)	Length (bp)	%TEs[a]	%Genome[b]
LTR	59,800	18.44	23,359,366	23.81	5.13
LINE	30,151	9.30	16,460,776	16.78	3.61
SINE	657	0.20	73,625	0.08	0.02
DIRS	569	0.18	47,954	0.05	0.01
PLE	8,026	2.47	3,050,523	3.11	0.67
TIR	92,414	28.50	16,554,144	16.87	3.63
MITE	25,755	7.94	5,342,790	5.45	1.17
Helitron	99,356	30.64	28,943,139	29.50	6.35
Maverick	7,580	2.34	4,272,403	4.35	0.94

The data were obtained after manual curation of the TE library. [a]Corresponds to the percentage of annotated TEs relative to the total TE content. [b]The percentage refers to the contribution of each TE order to the entire *D. amaguana* genome.

Table S4. General overview of transposable element (TE) annotation in the *D. amaguana* genome by superfamily, including the number of copies, genome proportion (%), and total length (bp).

TE superfamilies	No. Copies	Copies (%)	Length (bp)	%TEs[a]	%Genome[b]
LTR[c]	21,070	6.50	7,398,829	7.54	1.62
LTR/Bel-Pao	2,836	0.87	1,119,826	1.14	0.25
LTR/Copia	689	0.21	416,493	0.42	0.09
LTR/Gypsy	25,029	7.72	12,246,253	12.48	2.69
LTR/LARD	7,562	2.33	1,614,099	1.65	0.35
LTR/TRIM	2,614	0.81	563,866	0.57	0.12
LINE[c]	12,763	3.94	9,932,727	10.12	2.18
LINE/CR1	5,922	1.83	2,791,896	2.85	0.61
LINE/I	988	0.30	426,266	0.43	0.09
LINE/Jockey	4,250	1.31	1,815,812	1.85	0.40
LINE/Loa	21	0.01	6,021	0.01	0.001
LINE/R1	6,020	1.86	1,372,118	1.40	0.30
LINE/R2	124	0.04	70,667	0.07	0.016
LINE/RTE	63	0.02	45,269	0.05	0.010
SINE[c]	657	0.20	73,625	0.08	0.02
DIRS[c]	569	0.18	47,954	0.05	0.01
PLE[c]	8,026	2.47	3,050,523	3.11	0.67
TIR[c]	72,667	22.41	12,441,417	12.68	2.73
TIR/CMC	783	0.24	51,819	0.05	0.01
TIR/MULE-NOF	3	0.00	216	0.0002	0.000
TIR/Merlin	89	0.03	19,550	0.02	0.004
TIR/P	839	0.26	90,048	0.09	0.02
TIR/PiggyBac	169	0.05	60,824	0.06	0.013
TIR/Pogo	26	0.01	3,310	0.003	0.001
TIR/Tc1-Mariner	8,853	2.73	2,204,757	2.25	0.48
TIR/Transib	320	0.10	185,921	0.19	0.04
TIR/hAT	8,665	2.67	1,496,282	1.53	0.33
MITE[c]	25,755	7.94	5,342,790	5.45	1.17
Helitron	99,356	30.64	28,943,139	29.50	6.35
Maverick	7,580	2.34	4,272,403	4.35	0.94

The data were obtained after manual curation of the TE library. [a]Corresponds to the percentage calculated considering the total number of TEs annotated in the assembled *D. amaguana* genome. [b]The percentage refers to the contribution of each TE superfamily to the entire *D. amaguana* genome. [c]These TEs could not be classified at a level deeper than the order level and therefore do not correspond to specific superfamilies.

Table S5. Main characteristics of putative satDNAs identified in the assembled *D. amaguana* genome.

satDNA family	Satellite confidence	Genome proportion (%)	Satellite probability	%AT	Repeat Unit Length (bp)
DamaSat01-97	High	1.75	0.889	76.29	97
DamaSat02-14	Low	0.97	0.149	57.14	14
DamaSat03-15	Low	0.78	0.032	60.00	15
DamaSat04-6	Low	0.43	0.220	66.67	6
DamaSat05-3461	High	0.18	0.979	50.97	3461
DamaSat06-184	Low	0.14	0.487	54.35	184
DamaSat07-154	Low	0.13	0.273	69.48	154
DamaSat08-7	Low	0.12	0.589	57.14	7
DamaSat09-6	Low	0.11	0.019	66.67	6
DamaSat10-566	Low	0.08	0.418	59.19	566
DamaSat11-6402	High	0.06	0.979	46.33	6402
DamaSat12-303	Low	0.05	0.046	69.31	303
DamaSat13-150	Low	0.04	0.602	66.67	150
DamaSat14-108	Low	0.04	0.902	62.04	108
DamaSat15-965	Low	0.02	0.132	61.21	965
DamaSat16-1260	Low	0.01	0.033	50.24	1260

Results were obtained using the RepeatExplorer pipeline from Illumina raw reads representing 0.5x genome coverage.
