# Supplementary material for: Identification of transposable elements and satellite DNA in the Neotropical species Drosophila amaguana from the Ecuadorian Andean Forests
Source: PLoS One. 2025 Dec 10;20(12):e0337390. doi: 10.1371/journal.pone.0337390 (PMC12694884; doi:10.1371/journal.pone.0337390)

Additional File 3: Supplementary Figures

Fig S1. Raw estimated TE content by class within the *D. amaguana* assembled genome. Each panel shows the results from a different de novo TE annotation tool using non-curated libraries: **(A)** EDTA, **(B)** RepeatModeler, and **(C)** reasonaTE. The doughnut charts represent the proportion of retrotransposons and DNA transposons in the genome, while the bar plots show the relative abundance of TE orders contributing to the total masked content. Only TE orders contributing ≥0.1% of the genome are displayed. Colors correspond to TE orders and are consistent across methods.

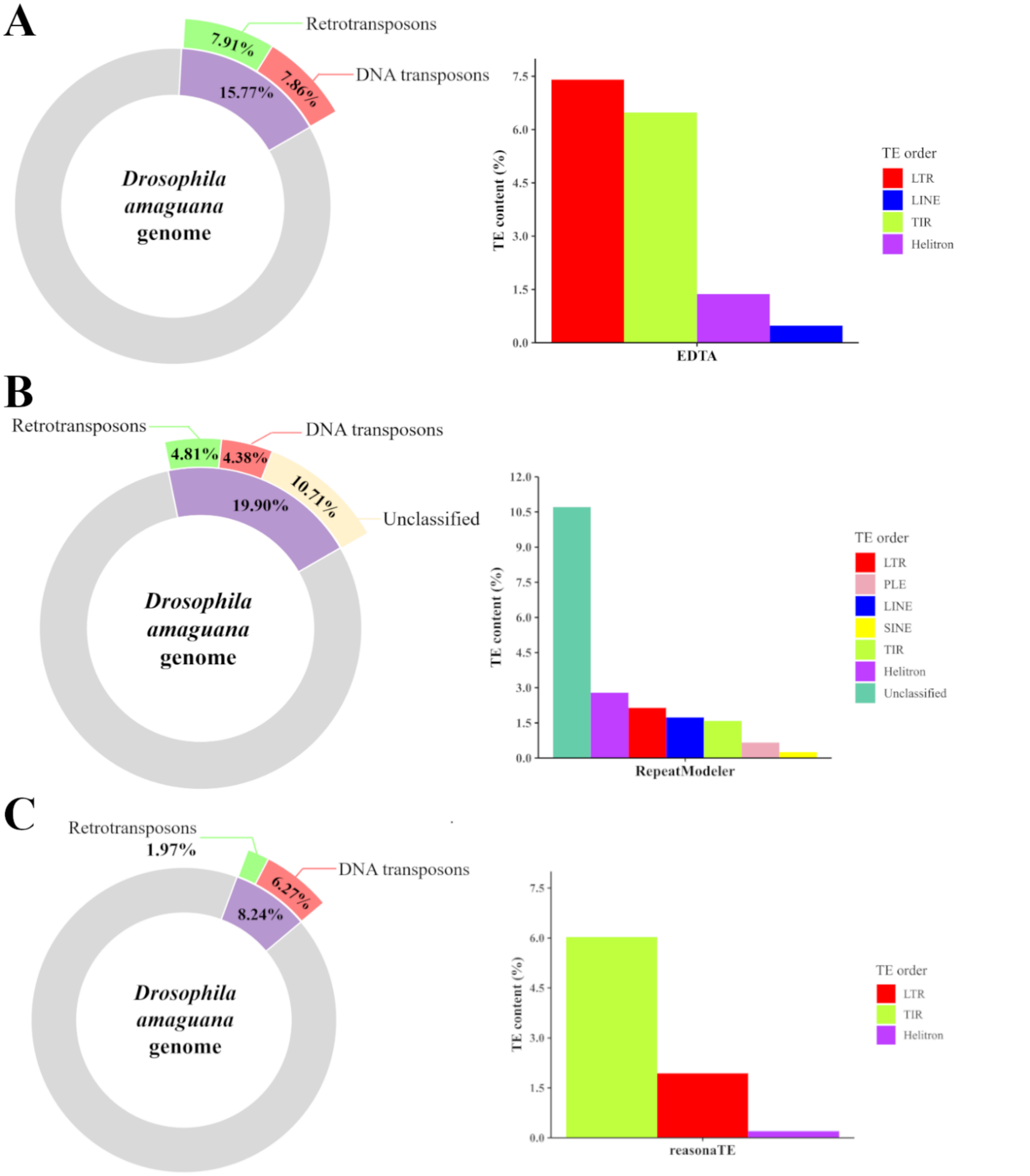

Fig S2. Size distribution of TE copies in *D. amaguana*. The boxplot shows the size distribution (in base pairs, bp) of all annotated transposable element (TE) copies for each superfamily in the assembled *D. amaguana* genome. Superfamily names are shown on the y-axis, and copy sizes on the x-axis. Each box spans from the 25th (Q1) to the 75th (Q3) percentile, with the line inside showing the median. Whiskers extend to 1.5 times the interquartile range (IQR), and outliers are omitted. Minor x-axis ticks aid in visualizing size variation.

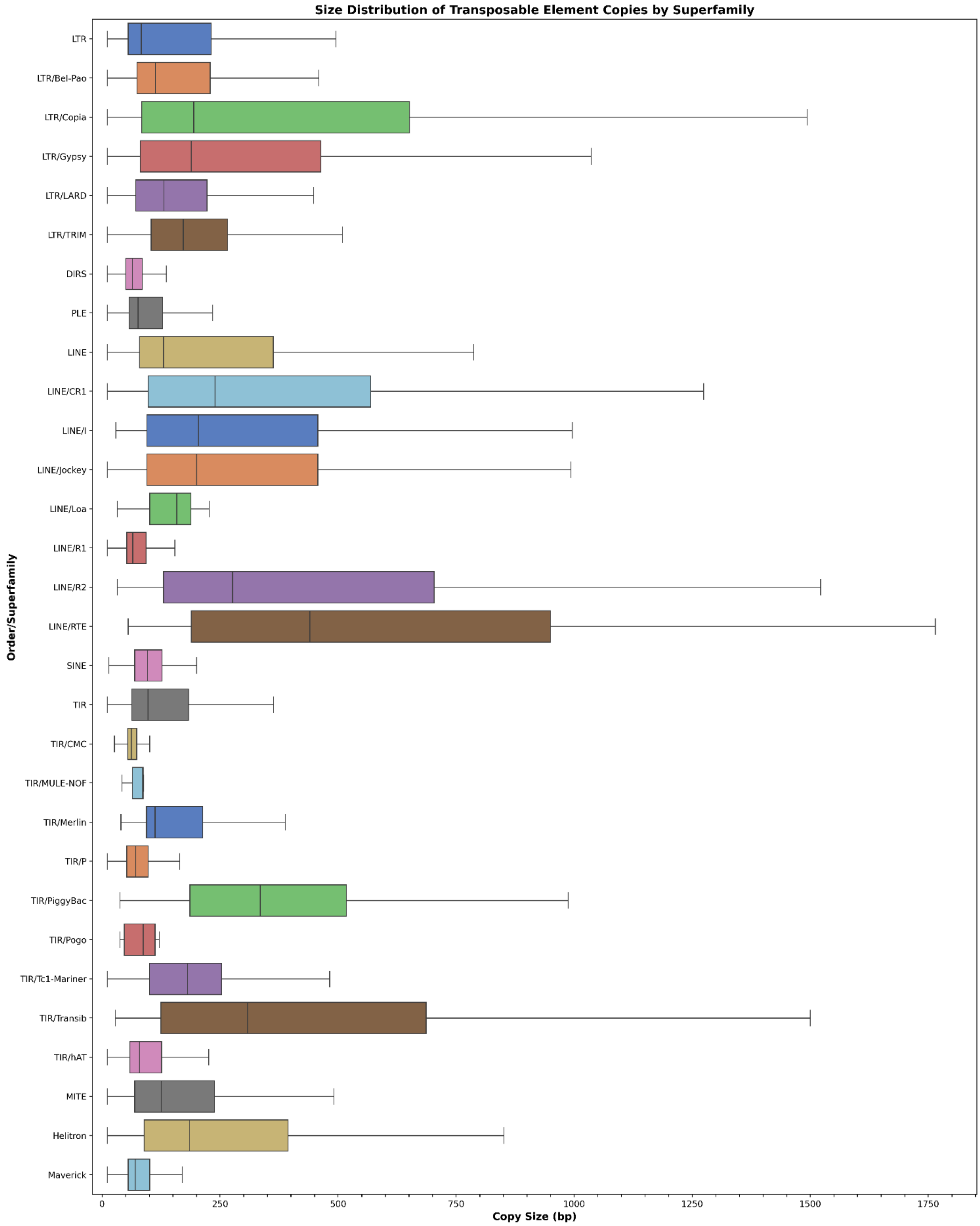

Fig S3. Outliers copy size distribution of TE copies in *D. amaguana*. This scatterplot shows the size (in base pairs, bp) of transposable element (TE) copies identified as outliers in Fig S2 -that is, those beyond 1.5 times the interquartile range (IQR)- for each superfamily in the assembled *D. amaguana* genome. Superfamily names are on the y-axis, and copy sizes on the x-axis. Each dot represents a single outlier TE copy. Minor x-axis ticks improve visualization of size variation.

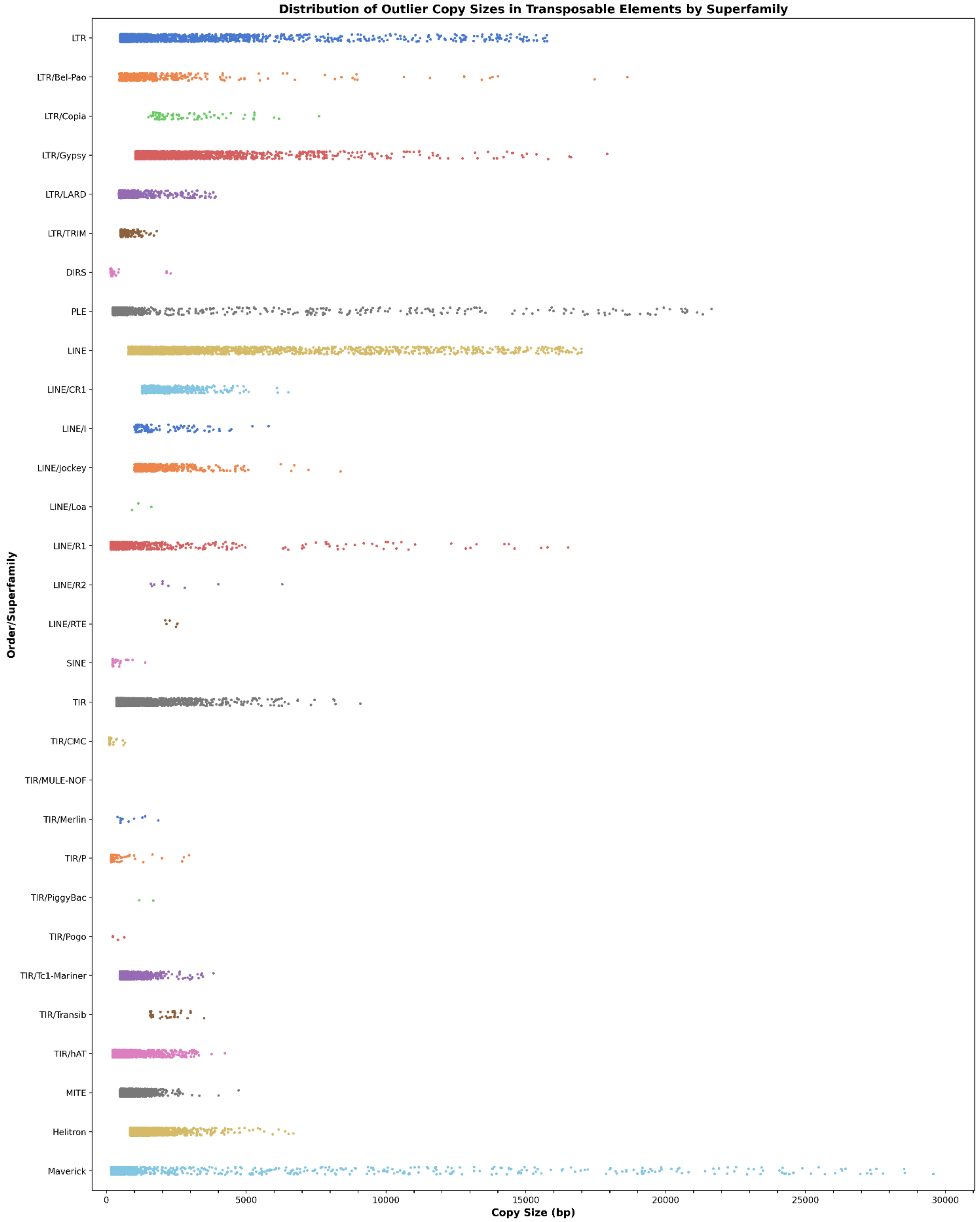

Supplement: S3 File — (PDF) [file pone.0337390.s003.pdf]
